# Immune-mediated comorbidities in multiple sclerosis: a scoping review

**DOI:** 10.3389/fimmu.2026.1911169

**Published:** 2026-10-28

**Authors:** Austin Daniel Solak, Patricia Mariana Riccio, Da Hee Seo, Samin Ayromlou, Azhar Mohammed Alanazi, Juan Manuel Racosta

**Affiliations:** 1 Department of Medicine, Western University Schulich School of Medicine & Dentistry, London, ON, Canada; 2 Department of Clinical Neurological Sciences, Western University Schulich School of Medicine & Dentistry, London, ON, Canada; 3 Department of Clinical Neurological Sciences, London Health Sciences Centre, London, ON, Canada; 4 Department of Clinical Neurological Sciences, London Health Sciences Centre Research Institute, London, ON, Canada

**Keywords:** autoimmunity, disability, disease progression, immune-mediated comorbidities, multiple sclerosis

## Abstract

**Background:**

Immune-mediated comorbidities are increasingly recognized in people with multiple sclerosis (PwMS), but their frequency, recorded diagnostic sequence, and clinical relevance remain incompletely characterized. We aimed to characterize their co-occurrence with MS, diagnoses following MS, associations with subsequently diagnosed MS, and relationships with MS clinical outcomes.

**Methods:**

We conducted a scoping review guided by the PRISMA-ScR checklist. PubMed, Embase, and Scopus were searched for studies published from January 1, 2001, through February 28, 2026. Eligible studies evaluated co-occurrence; immune-mediated diseases diagnosed following MS; associations between previously diagnosed immune-mediated diseases and subsequently diagnosed MS; or associations with MS activity, disability, or progression. Additional studies were identified through backward reference screening without publication-year restrictions.

**Results:**

Autoimmune thyroid disease, type 1 diabetes (T1D), psoriasis, inflammatory bowel disease (IBD), and bullous pemphigoid showed some of the most recurrent evidence of higher co-occurrence with MS, whereas findings for rheumatoid arthritis (RA), systemic lupus erythematosus (SLE), vitiligo, atopic dermatitis, asthma, and less frequently studied conditions were more variable. Studies investigating immune-mediated diseases diagnosed following MS reported heterogeneous, condition-specific associations, with higher subsequent occurrence for psoriasis, IBD, and bullous pemphigoid in some populations. Studies examining previously diagnosed immune-mediated diseases and subsequently diagnosed MS reported higher estimates for selected conditions, including T1D, psoriasis, IBD, bullous pemphigoid, systemic sclerosis, and primary Sjögren’s syndrome, although their magnitude, precision, and consistency varied. Evidence regarding associations between immune-mediated comorbidities and MS activity, disability, or progression was limited and heterogeneous. A small number of studies indicated adverse disability or MRI outcomes associated with T1D, whereas findings for other conditions were mixed, outcome-specific, or inconclusive.

**Conclusion:**

Several immune-mediated diseases demonstrated recurrent co-occurrence with MS, while separate studies reported condition-specific associations with diagnoses following MS or with subsequently diagnosed MS. Findings varied across diseases, study designs, and temporal frameworks, and may indicate shared susceptibility, surveillance bias, diagnostic delay, treatment exposure, residual confounding, or coincidental coexistence. Current evidence is insufficient to establish an MS prodrome or biologically distinct MS subgroups. Longitudinal studies using standardized disease definitions, documented diagnostic chronology, treatment data, and appropriate confounder adjustment are needed to clarify clinical and prognostic relevance.

## 1 Introduction

Multiple sclerosis (MS) is a chronic immune-mediated disease affecting approximately 2.8 million people worldwide and over 71,000 individuals in Canada (1, 2). Although its precise etiology remains incompletely understood, MS is widely recognized to arise from complex interactions among genetic susceptibility, environmental exposures, and dysregulated immune responses (3). Traditionally, MS has been viewed primarily as a central nervous system (CNS)-restricted autoimmune disease characterized by inflammatory demyelination and neurodegeneration. However, increasing evidence suggests that MS may instead represent one manifestation within a broader spectrum of systemic immune dysregulation.

Immune-mediated comorbidities are increasingly recognized in people with MS (PwMS). Several autoimmune and inflammatory diseases, including autoimmune thyroid disease, inflammatory bowel disease (IBD), psoriasis, rheumatoid arthritis (RA), systemic lupus erythematosus (SLE), and type 1 diabetes (T1D), have been reported at higher prevalence in PwMS than in the general population (4–6). However, reported associations differ considerably across various conditions and studies. Epidemiological co-occurrence alone is insufficient to determine whether these associations result from shared genetic or immunological susceptibility, common environmental factors, surveillance effects, treatment exposures, residual confounding, or coincidental coexistence.

The temporal relationship between immune-mediated diseases and MS may provide additional insight into these associations. Population-based studies have reported a higher occurrence of subsequently diagnosed MS among individuals with previously diagnosed T1D, psoriasis, and IBD (7–9). Although the MS prodromal period and broader preclinical changes have been discussed in the literature (10, 11), recorded diagnostic order does not establish that an immune-mediated condition preceded the biological or symptomatic onset of MS or represents a prodromal or causal factor. In addition, immune-mediated comorbidities may influence disease course, with some studies suggesting associations with greater disability progression independent of relapse activity (12, 13).

In this scoping review, we aimed to: (1) describe the co-occurrence of MS and immune-mediated conditions; (2) evaluate the occurrence of immune-mediated diseases following recorded MS diagnosis; (3) evaluate associations between previously diagnosed immune-mediated diseases and subsequently diagnosed MS; and (4) assess associations between immune-mediated comorbidities and MS activity, progression, and disability outcomes.

## 2 Methods

This scoping review was conducted to explore the breadth, scope, and characteristics of the literature on immune-mediated comorbidities in MS. The review followed the Preferred Reporting Items for Systematic Reviews and Meta-Analyses extension for Scoping Reviews (PRISMA-ScR) guidelines. No protocol was preregistered.

### 2.1 Eligibility criteria

Studies were eligible for inclusion if they included individuals with any subtype of MS or examined the occurrence of subsequently diagnosed MS among individuals with a previously diagnosed immune-mediated condition. Eligible studies were required to address at least one of the following objectives: (a) co-occurrence of MS and an immune-mediated condition; (b) occurrence of an immune-mediated condition following recorded MS diagnosis; (c) associations between previously diagnosed immune-mediated diseases and subsequently diagnosed MS; or (d) associations between immune-mediated comorbidities and MS disease activity, progression, or disability outcomes.

In this review, immune-mediated comorbidities were defined as conditions in which dysregulated innate and/or adaptive immune responses contribute centrally to disease pathogenesis, encompassing autoimmune, immune-mediated inflammatory, and atopic/allergic conditions. The immune-mediated comorbidities included in this review were autoimmune thyroid disease, T1D, psoriasis, IBD, RA, SLE, vitiligo, atopic dermatitis/eczema, asthma, celiac disease, bullous pemphigoid, primary Sjögren’s syndrome, systemic sclerosis/scleroderma, and uveitis. These conditions were grouped under a broad operational category of immune-mediated comorbidities to organize the epidemiological and clinical evidence. This grouping was not intended to imply that the conditions share a unified biological mechanism or immunopathogenic pathway.

We excluded reviews, meta-analyses, editorials/commentaries, case reports, small case series without a comparator, and genetic or Mendelian randomization studies as primary studies unless they reported original epidemiologic data relevant to MS risk. Studies examining other neurological conditions were excluded from comparisons unless MS-specific findings could be extracted separately.

### 2.2 Search strategy

PubMed, Embase, and Scopus were each searched on February 28, 2026, for English-language studies published from January 1, 2001, through February 28, 2026. The pragmatic lower boundary for the electronic database searches was chosen for 2001 because it marked the publication of the first McDonald diagnostic criteria for MS, which introduced magnetic resonance imaging (MRI) into a standardized diagnostic framework (14). These three databases were selected for their broad coverage of biomedical and clinical literature relevant to MS and immune-mediated diseases.

The search strategy combined terms for multiple sclerosis (“multiple sclerosis” OR “disseminated sclerosis”) with terms for immune-mediated conditions (“autoimmune disease” OR “inflammatory bowel disease” OR “psoriasis” OR “rheumatoid arthritis” OR “systemic lupus erythematosus” OR “type 1 diabetes” OR “thyroid disease” OR “asthma” OR “atopic dermatitis” OR “vitiligo” OR “celiac disease”), and terms for epidemiologic or clinical outcomes (“comorbidity” OR “prevalence” OR “incidence” OR “epidemiology” OR “risk” OR “disease activity” OR “relapse” OR “disability” OR “progression” OR “MRI”). Database-specific search strategies, including controlled vocabulary and field restrictions, are provided in **Supplementary Table S1**.

The condition-specific search terms were not intended to constitute a closed list of eligible conditions. Additional immune-mediated conditions identified during literature screening were evaluated against the operational definition provided in Section 2.1. Eligibility decisions concerning conditions not individually named in the search strategy were made independently by two reviewers (ADS and JMR) during full-text screening and before data extraction. Disagreements were resolved through discussion and consensus before the condition was incorporated into the synthesis. Bullous pemphigoid, primary Sjögren’s syndrome, systemic sclerosis, and uveitis were identified through the broader “autoimmune disease” search term and backward reference-list screening. The same study-level eligibility criteria were applied to studies examining these conditions, irrespective of their reported findings. Because these additional conditions were not individually named in the electronic search strategy, their ascertainment may have been less comprehensive, and the possibility of selective ascertainment cannot be excluded.

Reference lists of eligible studies were screened to identify additional relevant articles not captured through the primary electronic database searches. Backward citation searching of eligible studies was not restricted by publication year, and studies published before 2001 were evaluated using the same eligibility criteria as studies identified through the primary database search described in Section 2.1. Because pre-2001 studies were identified through backward citation searching rather than a dedicated electronic database search, coverage of the eligible pre-2001 literature may be incomplete.

### 2.3 Study selection

Records retrieved from the three databases were exported to EndNote. The Find Duplicates function was configured to compare article title, author names, publication year, and journal name. Candidate duplicates were subsequently reviewed manually using DOI, PMID, and full bibliographic details where available. The database searches identified 1,568 records. After removing 398 duplicates, 1,170 unique database records remained. Backward reference screening identified 24 additional unique records, resulting in 1,194 records for title and abstract screening. Two reviewers (ADS and JMR) independently screened titles and abstracts according to the predefined eligibility criteria. Articles considered potentially eligible by either reviewer underwent full-text assessment. Both reviewers independently evaluated the full-text articles and recorded the primary reason for exclusion. Disagreements arising during title and abstract screening or full-text assessment were resolved through discussion and consensus. Articles identified through backward citation searching were evaluated using the same eligibility criteria and screening procedures. Of the 1,194 records screened, 934 were excluded, and 260 full-text articles were assessed for eligibility. Following the exclusion of 161 full-text articles, 99 studies met the eligibility criteria and were included in the final synthesis. Detailed study-level characteristics are provided in **Supplementary Table S2**.

### 2.4 Data extraction

Data were independently extracted by two reviewers (ADS and JMR) using a standardized data-extraction form. The independently completed forms were compared, and discrepancies were resolved through discussion and consensus. Extracted variables included study design, study population and sample size, comparator group, MS and comorbidity definitions and ascertainment methods, temporal framework, raw occurrence data, effect measure and estimate with 95% confidence interval (CI), principal adjustment variables, follow-up duration, relevant treatment exposure, and study-specific limitations.

For studies examining associations between immune-mediated comorbidities and MS clinical outcomes, the corresponding outcome definitions and numerical findings were also extracted, including measures of disease activity, relapse, disability, progression, and MRI outcomes, where available.

Definitions of immune-mediated comorbidities were reviewed for consistency across studies, while differences in diagnostic definitions and ascertainment methods were retained and considered when interpreting the findings. Where studies reported both adjusted and unadjusted estimates for the same comparison, adjusted estimates were preferentially presented in this review.

All quantitative values and directional interpretations used in the synthesis were subsequently checked against the original reports. Discrepancies identified during verification were reviewed against the source articles and resolved through discussion and consensus.

### 2.5 Evidence synthesis

Given heterogeneity in study design, populations, disease definitions, comparator groups, follow-up periods, and effect measures, findings were synthesized narratively rather than quantitatively pooled. Studies addressing the occurrence of immune-mediated comorbidities were organized according to three distinct epidemiological questions: (a) co-occurrence of MS and an immune-mediated condition when temporal sequence was not established or was not the primary analytic framework; (b) occurrence of an immune-mediated condition following recorded MS diagnosis; and (c) occurrence of MS following the recorded diagnosis of an immune-mediated condition. These analyses were presented separately because studies addressing different epidemiological questions are subject to different sources of bias.

Within each temporal framework, findings were descriptively grouped as Higher Occurrence, No Clear Difference, Lower Occurrence, Subgroup-specific, or Descriptive relative to the respective comparator. Categorization was based on the direction, magnitude, precision, and consistency of the reported comparative estimates, together with the comparator and outcome definition.

Studies were categorized as higher if the comparative evidence supported a greater occurrence of the outcome in the exposed group relative to the comparator. Studies were categorized as lower if the comparative evidence supported a lower occurrence of the outcome in the exposed group relative to the comparator. No clear difference was assigned when comparative estimates did not provide sufficiently clear evidence of either higher or lower occurrence, including near-null or imprecise estimates. Subgroup-specific findings were those in which materially different associations were observed across relevant subgroups or disease subtypes, such that assigning a single overall direction would be misleading. Descriptive findings included studies reporting counts, proportions, prevalence, or other occurrence measures without a usable disease-specific comparative effect estimate, as well as non-estimable results.

Studies examining associations between immune-mediated comorbidities and MS clinical outcomes were synthesized separately and grouped as Worse Outcomes, No Clear Difference, Better Outcomes, Subgroup-specific, or Descriptive. Worse Outcomes was assigned when comparative evidence supported an adverse association with at least one clinically relevant MS outcome and the remaining assessed outcomes showed no clear difference rather than an opposing favorable association. Conversely, Better Outcomes was assigned when comparative evidence supported a favorable association with at least one clinically relevant MS outcome, and the remaining assessed outcomes showed no clear difference. No Clear Difference was assigned when available comparative evidence did not consistently support either worse or better outcomes. Subgroup-specific was reserved for studies reporting materially different associations across demographic, clinical, or temporal subgroups. Studies lacking an appropriate comparator group or a usable disease-specific comparative analysis of MS outcome data were classified as Descriptive.

These categories served only as descriptive organizational labels and were not used for vote counting or grading the strength of evidence. Effect estimates were interpreted in conjunction with their 95% CIs, considering the magnitude, direction, precision, and methodological context of the reported associations. Statistical significance was not used as an independent criterion for assigning evidence categories. Where available, p-values were retained to describe the original studies’ findings, particularly for prevalence comparisons, subgroup analyses, and descriptive outcomes for which effect estimates or CIs were unavailable. However, p-values were interpreted alongside the magnitude and precision of the reported findings rather than used independently to determine the direction of an association.

## 3 Search results and overview of included studies

Our search identified 1,568 records from database searches, including 436 from PubMed, 612 from Embase, and 520 from Scopus. After removing 398 duplicates, 1,170 unique database records remained. Backward reference screening identified 24 additional unique records, resulting in 1,194 records for title and abstract screening. Of these, 934 were excluded, and 260 full-text articles were assessed for eligibility. Following the exclusion of 161 full-text articles, 99 studies met the eligibility criteria and were included in the final synthesis (**Figure 1**). These studies addressed four main questions: (a) the co-occurrence of MS and immune-mediated conditions, (b) the occurrence of immune-mediated diseases following recorded MS diagnosis, (c) associations between previously diagnosed immune-mediated diseases and subsequently diagnosed MS, and (d) associations between immune-mediated comorbidities and MS activity, disability, or progression.

**Figure 1:**
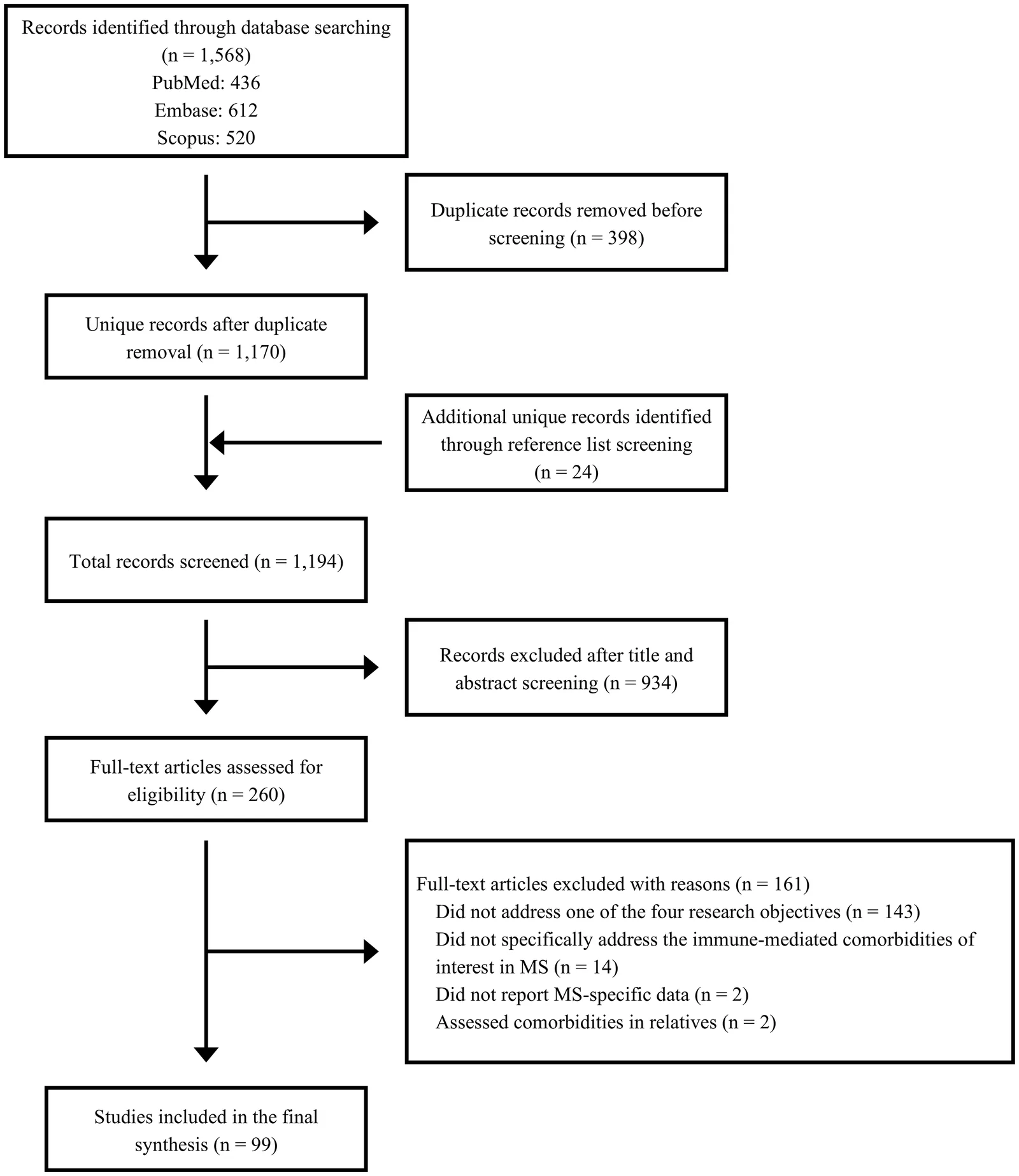
Flowchart of study selection. A total of 1,568 records were identified through database searches. Following the removal of 398 duplicates, 1,170 unique records remained. Backward reference screening identified an additional 24 unique records, resulting in 1,194 records for title and abstract screening. After excluding 934 records, 260 full-text articles were assessed for eligibility. Of these, 161 were excluded, and 99 studies were included in the final synthesis.

## 4 Co-occurrence of MS and immune-mediated comorbidities

Sixty-six unique studies examined the co-occurrence of MS and immune-mediated comorbidities (**Table 1**). Findings were heterogeneous across conditions and study designs. Autoimmune thyroid disease, T1D, psoriasis, IBD, and bullous pemphigoid showed more consistent evidence of higher co-occurrence, whereas findings for RA, SLE, vitiligo, atopic dermatitis/eczema, asthma, celiac disease, primary Sjögren’s syndrome, and systemic sclerosis were more variable. Studies that reported only case counts, prevalence estimates, or raw comparisons without an appropriate comparative effect estimate were retained as descriptive evidence. Importantly, the studies discussed in this section describe epidemiological co-occurrence but do not establish a temporal sequence, causality, or a shared biological mechanism between MS and the comorbid conditions.

**Table 1:** Co-occurrence of multiple sclerosis and immune-mediated comorbidities.

Categorization	Reference(s)	Findings
Autoimmune thyroid disease / thyroid disease		
Higher Occurrence	García-Alvarado 2026 (25)	Graves’: OR 4.48 (3.24–6.20) Hypothyroidism: OR 3.32 (3.08–3.59)
	Gnavi 2025 (26)	Hashimoto’s: OR 1.61 (1.41–1.83) Hypothyroidism: OR 1.32 (1.20–1.45)
	Kang 2010 (27)	Hypothyroidism: OR 3.20 (1.7–6.1)
	Munteis 2007 (28)	Hypothyroidism: OR 3.00 (1.10–8.60) ATA Positive: OR 5.25 (2.16–12.20)
	Edwards and Constantinescu 2004 (29)	OR 1.8 (1.07–3.02)
No Clear Difference	Farez 2014 (30)	OR 1.59 (0.8–3.3)
	Boelaert 2010 (31)	Graves’: Female RR 1.38 (0.60–2.72) Hashimoto’s: Female RR 2.81 (0.58–8.16)
	Langer-Gould 2010 (32)	Graves’: OR 0.7 (0.4–1.3) Hashimoto’s: OR 1.0 (0.5–1.9)
	Ramagopalan 2007 (33)	OR 1.14 (0.72–1.82)
	Midgard 1996 (34)	Goiter: OR 5.27 (0.51–261)
Lower Occurrence	—	—
Descriptive	Leal Rato 2023 (15)	2.0%
	Wawrzyniak 2023 (16)	Hashimoto’s: 3.67% Graves’: 0.52%
	Dai 2022 (17)	16.7%
	Ciampi 2020 (18)	15.7%
	Ershadinia 2020 (19)	Hashimoto’s: 2.47% Graves’: 0.25%
	Kapica-Topczewska 2020 (24)	Lower prevalence in MS vs controls; numerical comparison reported graphically
	Ben Achour 2017 (20)	Hypothyroidism: 4.76%
	Deretzi 2015 (21)	5.19% vs 3.29%, *p* = 0.009
	Laroni 2006 (22)	Graves’: 2.04% vs 1.22%
	Henderson 2000 (23)	Hypothyroidism: 2.56% vs 3.60%
Subgroup-specific	Marrie 2012 (35)	Age 20–44: PR 1.25 (1.15–1.37) Age 45–59: PR 1.25 (1.17–1.33) Age 60+: PR 0.88 (0.85–0.92)
	Sloka 2005 (37)	Graves’: 3.1% vs 0.4%, *p* = 0.002 Hashimoto’s: 5.5% vs 2.2%, *p* = 0.097
	Niederwieser 2003 (36)	Male: 9.4% vs 1.9%, *p* = 0.03 Female: 8.7% vs 9.2%, *p* = 0.87
Type 1 diabetes		
Higher Occurrence	García-Alvarado 2026 (25)	OR 2.92 (2.44–3.50)
	Gnavi 2025 (26)	OR 1.54 (1.22–1.93)
	Murtonen 2018 (38)	OR 2.11 (1.32–3.36)
	Bechtold 2014 (9)	RR 3.35–4.79 (1.56–7.21 and 2.01–11.39)
	Roshanisefat 2012 (39)	HR 1.30 (1.22–1.40)
	Edwards and Constantinescu 2004 (29)	OR 18.14 (7.97–41.30)
	Marrosu 2002 (40)	OR 2.99 (1.77–5.04)
No Clear Difference	Lo 2021 (41)	PR 0.97 (0.58–1.61)
	Tettey 2016 (42)	PR 2.01 (0.23–7.27)
	Farez 2014 (30)	OR 1.00 (0.3–3.4)
	Langer-Gould 2010 (32)	OR 0.9 (0.7–1.3)
	Ramagopalan 2007 (33)	OR 1.14 (0.72–1.82)
Lower Occurrence	—	—
Descriptive	Leal Rato 2023 (15)	1.7%
	Wawrzyniak 2023 (16)	0.26%
	Ershadinia 2020 (19)	0.16%
	Ben Achour 2017 (20)	14.29%
	Deretzi 2015 (21)	0.51% vs 0.38%, *p* = 0.549
	Kang 2010 (27)	0.3% vs 0.0%
	Hussein and Reddy 2006 (43)	0.92% (0.38–1.46)
	Laroni 2006 (22)	3.67% vs 0.41%, OR 9.3, *p* = 0.02
	Henderson 2000 (23)	0.85% vs 0.9%
	Wertman 1992 (44)	0.898% vs 0.01%
Subgroup-specific	—	—
Psoriasis		
Higher Occurrence	García-Alvarado 2026 (25)	OR 2.11 (1.73–2.56)
	Lo 2021 (41)	PR 2.41 (2.04–2.85)
	Guido 2017 (46)	OR 1.52 (1.01–2.29)
	Marrie 2017 (45)	PR 1.31 (1.25–1.37)
	Tettey 2016 (42)	PR 2.65 (1.48–4.38)
	Fellner 2014 (47)	OR 8.39 (1.05–66.81)
	Roshanisefat 2012 (39)	HR 1.57 (1.29–1.91)
	Edwards and Constantinescu 2004 (29)	OR 2.11 (1.13–3.94)
No Clear Difference	Esfandiari 2023 (48)	OR 4.63 (0.35–60.6)
	Farez 2014 (30)	OR 0.13 (0–1.1)
	Langer-Gould 2010 (32)	OR 1.1 (0.8–1.4)
	Ramagopalan 2007 (33)	OR 1.06 (0.78–1.44)
	Midgard 1996 (34)	OR 2.01 (0.73–5.83)
Lower Occurrence	—	—
Descriptive	Mangkorntongsakul 2025 (49)	13.7%
	Leal Rato 2023 (15)	0.9%
	Wawrzyniak 2023 (16)	0.26%
	Ershadinia 2020 (19)	0.25%
	Deretzi 2015 (21)	1.03% vs 0.57%
	Laroni 2006 (22)	0.41% vs 0.82%
	Henderson 2000 (23)	2.56% vs 0%
Subgroup-specific	—	—
Inflammatory bowel disease		
Higher Occurrence	García-Alvarado 2026 (25)	Overall: OR 2.94 (2.33–3.71) Crohn’s: OR 3.49 (2.62–4.65) UC: OR 2.49 (1.68–3.69)
	Roshanisefat 2012 (39)	Crohn’s: HR 1.45 (1.16–1.80) UC: HR 1.43 (1.11–1.83)
	Langer-Gould 2010 (32)	OR 1.7 (1.2–2.5)
	Gupta 2005 (50)	Overall: OR 1.44 (1.07–1.94) Crohn’s: OR 1.35 (0.83–2.22) UC: OR 1.49 (1.03–2.16)
	Edwards and Constantinescu 2004 (29)	Crohn’s: OR 3.17 (1.01–9.95) UC: OR 3.15 (1.30–7.64)
No Clear Difference	Tettey 2016 (42)	Crohn’s: PR 3.6 (0.51–25.4) UC: PR 2.97 (0.42–20.98)
	Farez 2014 (30)	Crohn’s: OR 1.00 (0.1–16)
	Ramagopalan 2007 (33)	Crohn’s: OR 0.75 (0.07–8.23) UC: OR 2.20 (0.37–13.42)
Lower Occurrence	—	—
Descriptive	Leal Rato 2023 (15)	0.9%
	Pérez Díaz del Campo 2023 (53)	0.24% vs 0.18%
	Wawrzyniak 2023 (16)	0%
	Dai 2022 (17)	1.70%
	Deretzi 2015 (21)	0.42% vs 0.57%
	Henderson 2000 (23)	2.56% vs 0%
	Pokorny 2007 (54)	0.4/100,000 vs 0.0472 / 100,000
	Tremlett 2002 (55)	OR inf (1.30–inf)
Subgroup-specific	Magyari 2014 (11)	UC / Male: OR 2.22 (0.93–4.59) UC / Female: no clear difference
	Bernstein 2005 (51)	Crohn’s: PR 1.11 (0.67–1.84) UC: PR 1.9 (1.19–3.03)
Rheumatoid arthritis		
Higher Occurrence	García-Alvarado 2026 (25)	OR 1.77 (1.30–2.41)
	Kang 2010 (27)	OR 4.80 (2.9–8.1)
No Clear Difference	Gnavi 2025 (26)	OR 0.93 (0.71–1.22)
	Lo 2021 (41)	PR 0.86 (0.64–1.16)
	Tettey 2016 (42)	PR 0.69 (0.19–1.76)
	Farez 2014 (30)	OR 0.30 (0–3.2)
	Langer-Gould 2010 (32)	OR 1 (0.7–1.3)
	Ramagopalan 2007 (33)	OR 1.21 (0.75–1.98)
	Edwards and Constantinescu 2004 (29)	OR 0.93 (0.23–3.74)
	Tremlett 2002 (55)	OR 1.25 (0.36–4.30)
Lower Occurrence	Żur-Wyrozumska 2026 (56)	PR 0.57 (0.51–0.64)
	Roshanisefat 2012 (39)	HR 0.77 (0.65–0.91)
Descriptive	Wawrzyniak 2023 (16)	0.52%
	Ershadinia 2020 (19)	0.66%
	Deretzi 2015 (21)	0.23% vs 0.25%
	Laroni 2006 (22)	0.82% vs 0.82%
	Henderson 2000 (23)	3.42% vs 0.45%
	Midgard 1996 (34)	1.94% vs 0%
Subgroup-specific	—	—
Systemic lupus erythematosus		
Higher Occurrence	García-Alvarado 2026 (25)	OR 5.21 (3.89–6.97)
	Kang 2010 (27)	OR 26.90 (10.3–70.3)
No Clear Difference	Gnavi 2025 (26)	OR 1.33 (0.79–2.23)
	Langer-Gould 2010 (32)	OR 1.3 (0.8–2.2)
	Ramagopalan 2007 (33)	OR 0.75 (0.07–8.23)
Lower Occurrence	—	—
Descriptive	Leal Rato 2023 (15)	0.6%
	Wawrzyniak 2023 (16)	0%
	Ershadinia 2020 (19)	0.41%
	Deretzi 2015 (21)	0.23% vs 0.25%
	Fanouriakis 2014 (58)	9 overlap cases; Prevalence 1.0–1.2% in the SLE and MS cohorts
	Henderson 2000 (23)	1.71% vs 0.45%
Subgroup-specific	—	—
Vitiligo		
Higher Occurrence	García-Alvarado 2026 (25)	OR 2.46 (1.57–3.87)
No Clear Difference	Langer-Gould 2010 (32)	OR 0.9 (0.3–2.3)
	Ramagopalan 2007 (33)	OR 0.64 (0.16–2.47)
Lower Occurrence	—	—
Descriptive	Kavyani 2024 (60)	Prevalence 2.12% (1.06–3.17)
	Deretzi 2015 (21)	0.65% vs 0.06%
	Laroni 2006 (22)	0.41% vs 1.22%
	Henderson 2000 (23)	0% vs 0.90%
Subgroup-specific	—	—
Atopic dermatitis		
Higher Occurrence	Ivert 2021 (61)	OR 1.28 (1.15–1.42)
	Tettey 2016 (42)	Eczema: PR 26.2 (17.11–38.38)
No Clear Difference	García-Alvarado 2026 (25)	OR 1.15 (0.94–1.41)
	Farez 2014 (30)	OR 0.54 (0.2–1.2)
	Roshanisefat 2012 (39)	Eczema: HR 0.99 (0.65–1.53)
	Langer-Gould 2010 (32)	OR 1 (0.9–1.2)
	Edwards and Constantinescu 2004 (29)	Eczema: OR 0.81 (0.49–1.36)
	Tremlett 2002 (55)	Eczema: OR 0.67 (0.37–1.20)
Lower Occurrence	—	—
Descriptive	Wawrzyniak 2023 (16)	0.26%
	Karimi 2013 (62)	Eczema: 7.5% vs 17.5%, *p* = 0.193
Subgroup-specific	—	—
Asthma		
Higher Occurrence	Hill 2019 (63)	PR 2.97 (2.96–2.97)
	Tettey 2016 (42)	PR 1.85 (1.28–2.58)
	Edwards and Constantinescu 2004 (29)	OR 2.86 (2.14–3.82)
No Clear Difference	Briggs 2026 (64)	OR 0.78 (0.58–1.03)
	Bourne 2017 (65)	OR 1.04 (0.66–1.66)
	Manouchehrinia 2015 (66)	OR 0.89 (0.68–1.17)
	Farez 2014 (30)	OR 0.50 (0.2–1)
	Langer-Gould 2010 (32)	OR 0.9 (0.8–1.1)
	Ponsonby 2006 (67)	OR 1.67 (1.00–2.80)
Lower Occurrence	Tremlett 2002 (55)	OR 0.33 (0.15–0.77)
Descriptive	Dai 2022 (17)	6.2%
	Ben Achour 2017 (20)	4.76%
	Karimi 2013 (62)	2.5% vs 2.5%
Subgroup-specific	—	—
Celiac disease		
Higher Occurrence	Gnavi 2025 (26)	OR 2.01 (1.56–2.59)
	Tettey 2016 (42)	PR 5.05 (1.27–20.05)
	Rodrigo 2011 (68)	tTG-IgA positive: OR 5.33 (1.07–26.43)
No Clear Difference	Farez 2014 (30)	OR 0.33 (0–3.2)
	Edwards and Constantinescu 2004 (29)	OR 0.37 (0.11–1.30)
Lower Occurrence	—	—
Descriptive	Wawrzyniak 2023 (16)	1.05%
	Piccini 2020 (70)	0.6% vs 1%, *p* = 0.65
	de Oliveira 2016 (71)	0.40%
	Khoshbaten 2012 (72)	Anti-tTGA positive: 0% vs 0%
	Nicoletti 2008 (73)	CD-related serology: 0% vs 0.5%
	Borhani Haghighi 2007 (74)	IgA AGA: 3.1% vs 3.6% IgG AGA: 3.7% vs 3.0%
	Salvatore 2004 (75)	tTG positive: 0% vs 0.57%
Subgroup-specific	Shor 2009 (69)	IgG AGA: OR 5.3 (1.08–26.13) tTG-IgG: OR 13.4 (0.71–251.62)
Bullous pemphigoid		
Higher Occurrence	Kibsgaard 2017 (76)	OR 9.7 (6.0–15.6)
	Roshanisefat 2012 (39)	HR 9.42 (5.84–15.20)
	Langer-Gould 2010 (32)	OR 6.7 (1.5–29.9)
No Clear Difference	—	—
Lower Occurrence	—	—
Descriptive	—	—
Subgroup-specific	—	—
Primary Sjögren’s syndrome		
Higher Occurrence	García-Alvarado 2026 (25)	OR 4.91 (3.59–6.71)
No Clear Difference	Gnavi 2025 (26)	OR 1.36 (0.74–2.47)
	Langer-Gould 2010 (32)	OR 2.0 (0.9–4.5)
Lower Occurrence	—	—
Descriptive	Leal Rato 2023 (15)	0.3%
	Hora 2022 (77)	1.49%
	Deretzi 2015 (21)	0% vs 0.19%
	Miró 1990 (78)	3.1%
	Noseworthy 1989 (79)	0%
Subgroup-specific	—	—
Systemic sclerosis		
Higher Occurrence	—	—
No Clear Difference	Langer-Gould 2010 (32)	OR 1 (0.3–3.4)
Lower Occurrence	—	—
Descriptive	—	—
Subgroup-specific	—	—
Uveitis		
Higher Occurrence	Langer-Gould 2010 (32)	OR 2.2 (1.7–2.9)
	Edwards and Constantinescu 2004 (29)	OR 65 (38–110)
No Clear Difference	Farez 2014 (30)	OR 2.02 (0.2–22)
Lower Occurrence	—	—
Descriptive	Leal Rato 2023 (15)	2/349 (0.6%)
	Laroni 2006 (22)	1/245 (0.41%)
Subgroup-specific	—	—

AGA, anti-gliadin antibody; CD, Celiac disease; HR, hazard ratio; IgA, immunoglobulin A; IgG, immunoglobulin G; OR, odds ratio; PR, prevalence ratio; RR, relative risk; tTG, tissue transglutaminase; tTGA, tissue transglutaminase antibody; UC, ulcerative colitis. Values in parentheses following effect estimates represent 95% confidence intervals unless otherwise specified.

### 4.1 Autoimmune thyroid disease

Autoimmune thyroid disease was among the most frequently reported immune-mediated comorbidities in PwMS, although prevalence estimates varied across study populations and specific thyroid diseases, including autoimmune thyroiditis, Graves’ disease, Hashimoto thyroiditis, and hypothyroidism. Across descriptive studies, thyroid disease prevalence in PwMS generally ranged from 2% to 17%, while estimates in general populations were typically lower, often ranging between 1% and 10% (15–20). Additional comparative studies reported prevalence differences for specific thyroid phenotypes, including autoimmune thyroid disease (5.19% vs 3.29%) (21), Graves’ disease (2.04 vs 1.22%) (22), and hypothyroidism (2.56% vs 3.60%) among PwMS and non-MS populations (23). A Polish population-based study also reported lower thyroid disease prevalence among PwMS than matched controls, although the numerical comparison was presented graphically rather than as a comparative effect estimate (24).

Several large comparative studies reported higher co-occurrence of autoimmune thyroid disease among PwMS (25–29). A Spanish population-based study reported higher odds ratio (OR) of Graves’ disease (OR 4.48, 95% CI 3.24–6.20), and hypothyroidism (OR 3.32, 95% CI 3.08–3.59) (25), while an Italian population-based study similarly reported higher odds of Hashimoto’s thyroiditis (adjusted OR 1.61, 95% CI 1.41–1.83) and hypothyroidism (adjusted OR 1.32, 95% CI 1.20–1.45) (26). Positive associations were also reported in Taiwanese, Spanish, and British cohorts (27–29).

Other studies yielded less consistent findings. Several investigations reported near-null associations or positive but imprecise estimates (30–34), such as an adjusted OR of 1.14 (95% CI 0.72–1.82) for autoimmune thyroid diseases (33), as well as large but highly imprecise estimates, including an OR of 5.27 (95% CI 0.51–261) for goiter (34). In addition, subgroup-specific findings varied by age, sex, and thyroid disease subtype. For example, a Canadian population-based study reported a higher prevalence ratio (PR) of 1.25 in both the 20–44 and 45–59-year age groups, but a lower PR of 0.88 (95% CI 0.85–0.92) among individuals aged ≥60 years (35). An Austrian study reported sex-specific findings with higher autoimmune thyroid disease prevalence among male PwMS than male controls (9.4% vs 1.9%, *p* = 0.03), but similar prevalence among females (8.7% vs 9.2%, *p* = 0.87) (36). A Canadian cohort reported differences by thyroid disease subtype among PwMS compared to controls: Graves’ disease (3.1% vs 0.4%, *p* = 0.002) and Hashimoto thyroiditis (5.5% vs 2.2%, *p* = 0.097) (37). Overall, thyroid disease showed recurrent evidence of higher co-occurrence with MS in several populations, but findings were not uniform across thyroid phenotypes, demographic subgroups, and study populations.

### 4.2 Type 1 diabetes

Several comparative studies reported higher co-occurrence of T1D among PwMS (9, 25, 26, 29, 38–40). A Spanish population-based study reported an OR of 2.92 (95% CI 2.44–3.50) (25), a Finnish population-based study reported an adjusted OR of 2.11 (95% CI 1.32–3.36) (38), and an earlier Italian familial study reported an OR of 2.99 (95% CI 1.77–5.04) compared with healthy siblings of PwMS (40).

However, other comparative studies produced estimates that did not clearly support higher or lower co-occurrence (30, 32, 33, 41, 42). For example, an Australian cohort reported an adjusted PR of 2.01 (95% CI 0.23–7.27) (42), while an American population-based study reported an adjusted OR of 0.9 (95% CI 0.7–1.3) (32). Additional descriptive studies lacking disease-specific comparative effect estimates reported T1D prevalence ranging from 0.16% to 14.29% among PwMS (15, 16, 19–23, 27, 43, 44). Some also reported observed prevalence comparisons with non-MS groups, including 0.51% vs 0.38% (21) and 0.85% vs 0.90% (23). Overall, comparative studies generally supported a higher co-occurrence of T1D and MS, although the magnitude and precision of estimates varied across study populations and designs.

### 4.3 Psoriasis

Psoriasis demonstrated relatively consistent evidence of higher co-occurrence with MS across several comparative studies. An Australian survey-based study reported an adjusted PR of 2.41 (95% CI 2.04–2.85) (41), while a Canadian population-based study similarly reported an adjusted PR of 1.31 (95% CI 1.25–1.37) (45). A large American single-center cohort yielded increased co-occurrence of MS and psoriasis (adjusted OR 1.52, 95% CI 1.01–2.29) (46), while a single-center case-control study from Israel supported higher psoriasis occurrence among PwMS (OR 8.39, 95% CI 1.05–66.81) (47). Additional studies from Spain, Australia, Sweden, and the UK generally supported higher psoriasis occurrence among PwMS compared to the comparator (25, 29, 39, 42).

However, other comparative studies did not provide clear evidence of either higher or lower co-occurrence (30, 32–34, 48). Estimates ranged from OR 0.13 (95% CI 0–1.1) (30) to strongly positive but highly imprecise estimates such as an adjusted OR of 4.63 (95% CI 0.35–60.6) reported by an Iranian case-control study (48). In addition, several studies presented descriptive prevalence data without comparative effect estimates. Reported psoriasis prevalence among PwMS ranged from 0.25% to 13.7% across cohorts (15, 16, 19, 49). Other studies reported descriptive comparisons between PwMS and non-MS matched groups, including 1.03% vs 0.57% (21), 0.41% vs 0.82% (22), and 2.56% vs 0% (23). Overall, psoriasis showed recurrent evidence of higher co-occurrence with MS in several comparative studies, although null findings and substantial variation in prevalence were also observed.

### 4.4 Inflammatory bowel disease

Several comparative studies reported higher co-occurrence of IBD, including Crohn’s disease and ulcerative colitis, among PwMS (25, 29, 32, 39, 50). A Spanish population-based study reported higher odds of Crohn’s disease (OR 3.49, 95% CI 2.62–4.65), ulcerative colitis (OR 2.49, 95% CI 1.68–3.69), and IBD overall (OR 2.94, 95% CI 2.33–3.71) (25). Similarly, a British matched cohort study reported a higher overall co-occurrence (OR 1.44, 95% CI 1.07–1.94), with disease-specific estimates of OR 1.35 (95% CI 0.83–2.22) for Crohn’s disease and OR 1.49 (95% CI 1.03–2.16) for ulcerative colitis (50).

Other studies produced less consistent findings. While several comparative studies reported positive point estimates for Crohn’s disease and ulcerative colitis, these were often imprecise and compatible with no clear difference (30, 33, 42). For example, an Australian study reported a PR of 3.6 (95% CI 0.51–25.4) for Crohn’s disease and a PR of 2.97 (95% CI 0.42–20.98) for ulcerative colitis (42). A Canadian population-based study demonstrated disease-specific heterogeneity, with no clear increase for Crohn’s disease (PR 1.11, 95% CI 0.67–1.84) but higher occurrence of ulcerative colitis (PR 1.90, 95% CI 1.19–3.03) (51). A Danish population-based study demonstrated sex-specific findings for ulcerative colitis over the full study period, with a higher point estimate among males (OR 2.22, 95% CI 0.93–4.59) but no corresponding association observed among females (11).

Additional studies provided descriptive evidence without a comparative effect estimate. The reported prevalence of IBD among PwMS ranged from 0% to 1.7% (15–17), while a meta-analysis estimated a pooled prevalence of IBD in PwMS at 0.6% (95% CI 0.4–0.9) (52). Observed comparisons from various studies indicated rates such as 0.24% vs 0.18% (53), 0.42% vs 0.57% (21), and 2.56% vs 0% (23). A retrospective study conducted in Australia reported an observed MS–ulcerative colitis co-occurrence rate of 0.4 per 100,000 compared with an expected rate of 0.0472 per 100,000 (54). Similarly, a British matched case-control study yielded a non-estimable OR with a lower confidence bound of 1.30 (55). Overall, comparative studies generally supported higher co-occurrence of IBD and MS, with the most consistent evidence observed for ulcerative colitis, although findings varied according to IBD subtype, study design, and population.

### 4.5 Rheumatoid arthritis

Evidence for RA was heterogeneous. Higher co-occurrence of MS and RA was reported in a Spanish population-based study (OR 1.77, 95% CI 1.30–2.41) (25), and a Taiwanese case-control study (adjusted OR 4.80, 95% CI 2.9–8.1) (27). Conversely, two large population-based studies from Sweden and Poland reported lower co-occurrence, with an adjusted hazard ratio (HR) of 0.77 (95% CI 0.65–0.91) and an adjusted PR of 0.57 (95% CI 0.51–0.64), respectively (39, 56).

Several additional studies reported estimates that were either near the null or too imprecise to support a clear increase or decrease in RA occurrence among PwMS (26, 29, 30, 32, 33, 41, 42, 55). Representative examples include adjusted estimates of OR 0.93 (95% CI 0.71–1.22) (26), PR 0.86 (95% CI 0.64–1.16) (41), and OR 1.21 (95% CI 0.75–1.98) (33). Other studies provided descriptive prevalence estimates or observed comparisons without comparative effect estimates. RA prevalence among PwMS ranged from 0.52% to 0.66% in Polish and Iranian cohorts (16, 19), while other studies reported 0.23% vs 0.25% (21), 0.82% vs 0.82% (22), 1.94% vs 0% (34), and 3.42% vs 0.45% (23). A broader synthesis of population-based MS studies estimated RA prevalence in PwMS at approximately 2.92% (6), which appears higher than the commonly cited general population prevalence of 0.5% to 1.0% reported in secondary evidence (57). Taken together, the available evidence did not support a consistent pattern of co-occurrence between RA and MS, with positive, inverse, and near-null associations reported across populations and study designs.

### 4.6 Systemic lupus erythematosus

Two comparative studies reported higher co-occurrence of SLE among PwMS, although the evidence base was relatively small. A Spanish population-based study reported substantially higher odds of SLE (OR 5.21, 95% CI 3.89–6.97) (25), while a Taiwanese case-control study observed an even larger positive association (adjusted OR 26.90, 95% CI 10.3–70.3) (27). Other comparative studies yielded positive but less precise estimates. An Italian population-based study reported an adjusted OR of 1.33 (95% CI 0.79–2.23) (26), while two other population-based studies conducted in the US and Canada also reported imprecise findings (32, 33).

Several cohorts contributed descriptive SLE data only. Reported SLE prevalence among PwMS ranged from 0% to 0.6% (15, 16, 19). Descriptive comparisons included 0.23% vs 0.25% (21) and 1.71% vs 0.45% (23). In addition, a Greek overlap cohort identified nine individuals with both MS and SLE, corresponding to approximately 1.0–1.2% of the respective MS and SLE cohorts (58). The available evidence suggests that SLE may be more frequent among PwMS in some populations; however, the number of comparative studies remains limited, and several estimates were imprecise.

### 4.7 Vitiligo

Vitiligo was less frequently reported as a comorbid immune-mediated condition in PwMS, and the findings were heterogeneous. A Spanish population-based study reported higher odds of vitiligo among PwMS (OR 2.46, 95% CI 1.57–3.87) (25). In contrast, adjusted effect estimates from two population-based studies conducted in the US (OR 0.9, 95% CI 0.3–2.3) and Canada (OR 0.64, 95% CI 0.16–2.47) did not provide clear evidence of a difference (32, 33). Consistent with these mixed findings, a meta-analysis of six case-control studies reported no clear overall association between MS and prevalent vitiligo (pooled OR 1.33, 95% CI 0.80–2.22) (59).

Descriptive evidence was similarly variable. A vitiligo cohort recruited from an Iranian clinic reported an MS prevalence of 2.12% (95% CI 1.06–3.17) (60), while other studies reported vitiligo occurrence of 0% vs 0.9% (23), 0.41% vs 1.22% (22), and 0.65% vs 0.06% (21), compared to respective controls. Collectively, the limited evidence did not establish a consistent conclusion on the association between vitiligo and MS.

### 4.8 Atopic dermatitis

Studies evaluating atopic dermatitis and eczema produced largely inconclusive findings, with most comparative studies reporting near-null or imprecise estimates and only a small number reporting higher co-occurrence among PwMS. Higher co-occurrence was reported in a Swedish population-based study of atopic dermatitis (adjusted OR 1.28, 95% CI 1.15–1.42) (61), and an Australian study assessing eczema (adjusted PR 26.2, 95% CI 17.11–38.38) (42).

However, several other comparative studies yielded estimates that were near the null or imprecise (25, 29, 30, 32, 39, 55). For example, an American population-based study reported an adjusted OR of 1.0 (95% CI 0.9–1.2) for atopic dermatitis (32), while another reported an OR of 0.81 (95% CI 0.49–1.36) for eczema (29). Additionally, two separate cohorts provided descriptive evidence. A Polish multicenter study reported atopic dermatitis in 0.26% of PwMS (16), while an American single-center cohort reported eczema in 7.5% of PwMS compared with 17.5% of matched controls (*p* = 0.193) (62). Overall, findings for atopic dermatitis and eczema were inconsistent, and variation in disease definitions further limited direct comparisons across studies.

### 4.9 Asthma

Asthma was commonly identified in PwMS, although the association with MS was mixed across available studies. Several comparative studies reported higher asthma co-occurrence among PwMS, including a large American population study (adjusted PR 2.97, 95% CI 2.96–2.97) (63), an Australian population-based cohort (adjusted PR 1.85, 95% CI 1.28–2.58) (42), and a British clinic-based case-control study (OR 2.86, 95% CI 2.14–3.82) (29). While these studies consistently suggested greater asthma occurrence among PwMS, a British case-control study with 320 PwMS and 320 matched controls reported a lower asthma co-occurrence (OR 0.33, 95% CI 0.15–0.77) (55).

Several other comparative investigations did not provide clear evidence of increased co-occurrence (30, 32, 64–67). An American multicenter case-control study reported a near-null estimate (adjusted OR 0.78, 95% CI 0.58–1.03) (64), while another American multicenter case-control study examining pediatric PwMS similarly observed little difference compared with healthy cohorts in the same centers (adjusted OR 1.04, 95% CI 0.66–1.66) (65). A British cohort study reported an imprecise estimate for asthma among PwMS (adjusted OR 0.89, 95% CI 0.68–1.17) (66), while an Australian population-based study reported a positive but imprecise estimate compared to matched controls (adjusted OR 1.67, 95% CI 1.00–2.80) (67).

Descriptive studies reported asthma prevalence of 6.2% in an American cohort (17) and 4.76% in a Tunisian pediatric cohort (20), while one Iranian case-control study observed similar asthma frequencies in PwMS and matched controls (2.5% in both groups) (62). Overall, asthma showed substantial heterogeneity across studies, with evidence of higher, lower, and uncertain co-occurrence depending on the population examined.

### 4.10 Celiac disease

Evidence regarding celiac disease was heterogeneous and complicated by substantial variation in disease definitions, with some studies evaluating biopsy-confirmed celiac disease and others assessing celiac-related serologic markers. Several comparative studies reported higher co-occurrence of celiac disease or celiac-related serology among PwMS. An Italian population-based study reported increased odds of celiac disease (adjusted OR 2.01, 95% CI 1.56–2.59) (26), while an Australian population-based study reported a higher prevalence ratio (PR 5.05, 95% CI 1.27–20.05) (42). Similarly, a Spanish prospective study reported higher tissue transglutaminase (tTG)-IgA positivity among PwMS (OR 5.33, 95% CI 1.07–26.43) (68).

In contrast, several studies did not provide clear evidence of increased co-occurrence. Single-center studies from the UK and Argentina reported imprecise estimates that were compatible with both increased and decreased occurrence (OR 0.37, 95% CI 0.11–1.30; OR 0.33, 95% CI 0–3.2) (29, 30). A comparative serology study conducted in Colombia demonstrated assay-specific findings, with higher antigliadin antibody (AGA)-IgG positivity (OR 5.3, 95% CI 1.08–26.13), while the estimate for tTG-IgG positivity was highly imprecise (OR 13.4, 95% CI 0.71–251.62) (69).

Additional studies contributed descriptive serologic or prevalence data only. Reported celiac disease prevalence among PwMS ranged from 0.4% to 1.1% (16, 70, 71), while several screening studies assessed tTG antibodies, antigliadin antibodies, or other celiac disease–related serologic markers and generally reported little difference between PwMS and comparator populations (72–75). Taken together, the available evidence for celiac disease and MS remains inconsistent. While several studies reported positive associations, many estimates were imprecise because of the rarity of confirmed celiac disease cases, and differences in diagnostic criteria and serologic assays limit direct comparison across studies.

### 4.11 Other immune-mediated comorbidities

Several additional immune-mediated conditions were reported in PwMS, although the available evidence remained limited. Bullous pemphigoid showed consistently higher co-occurrence in the three comparative studies identified. A Danish nationwide study reported an OR of 9.7 (95% CI 6.0–15.6) (76), a Swedish cohort reported an adjusted HR of 9.42 (95% CI 5.84–15.20) (39), and a US population-based study reported an adjusted OR of 6.7 (95% CI 1.5–29.9) (32). Although the relative estimates were large, bullous pemphigoid remained rare, and the magnitude of these estimates should be interpreted in the context of small case numbers.

Evidence for primary Sjögren’s syndrome was more heterogeneous. A Spanish population-based study reported higher odds (OR 4.91, 95% CI 3.59–6.71) (25), whereas estimates from an Italian cohort (adjusted OR 1.36, 95% CI 0.74–2.47) (26) and an American cohort (adjusted OR 2.0, 95% CI 0.9–4.5) (32) did not clearly establish a difference. Several additional studies reported primary Sjögren’s syndrome prevalence ranging from 0% to 3.1% among PwMS (15, 21, 77–79).

Systemic sclerosis was uncommon in the available studies. An American population-based study reported a comparable prevalence in PwMS and controls, with an identical prevalence of 0.06% in both groups (adjusted OR 1.0, 95% CI 0.3–3.4) (32).

Uveitis was also evaluated in several studies, with some comparative evidence suggesting higher co-occurrence among PwMS. A British prospective clinic-based study reported substantially higher odds of uveitis among PwMS compared with external population estimates (OR 65, 95% CI 38–110) (29), while an American population-based study similarly reported higher odds (adjusted OR 2.2, 95% CI 1.7–2.9) (32). In contrast, a case-control study from Argentina reported a positive but highly imprecise estimate (OR 2.02, 95% CI 0.2–22), providing no clear evidence of higher or lower co-occurrence (30). Additional descriptive studies reported uveitis in 0.6% of PwMS in a Portuguese cohort (15) and in 0.41% of PwMS from an Italian MS center (22).

Taken together, the available evidence suggests that certain less common immune-mediated diseases, particularly bullous pemphigoid and uveitis, may occur more frequently among PwMS. However, evidence for primary Sjögren’s syndrome and systemic sclerosis remains limited, and additional population-based studies are required to clarify these associations.

## 5 Associations between previously diagnosed MS and subsequently diagnosed immune-mediated diseases

Studies examining immune-mediated diseases diagnosed after MS were considered separately from co-occurrence studies because they address a distinct temporal question and may be influenced by surveillance, treatment exposure, and differential healthcare contact after MS diagnosis (**Table 2**). Across conditions, subsequent diagnoses of thyroid disease, psoriasis, IBD, RA, SLE, bullous pemphigoid, and primary Sjögren’s syndrome were reported more frequently in PwMS in at least some studies, whereas findings for T1D, vitiligo, atopic dermatitis/eczema, celiac disease, and systemic sclerosis were less consistent.

**Table 2:** Associations between previously diagnosed MS and subsequently diagnosed immune-mediated diseases.

Categorization	Reference(s)	Findings
Autoimmune thyroid disease / thyroid disease		
Higher Occurrence	Capkun 2015 (80)	ERR 1.50 (1.5–1.6)
	Somers 2009 (81)	SIR 144.4 (110–185)
No Clear Difference	Marrie 2012 (35)	IRR 0.98 (0.73–1.30)
Lower Occurrence	—	—
Descriptive	—	—
Subgroup-specific	Conrad 2023 (82)	Graves’: IRR 1.60 (1.3–1.8) Hashimoto’s: IRR 1.10 (1.0–1.2)
Type 1 diabetes		
Higher Occurrence	Roshanisefat 2012 (39)	HR 1.45 (1.34–1.57)
No Clear Difference	—	—
Lower Occurrence	—	—
Descriptive	Conrad 2023 (82)	IRR 0.00 (0.0–inf)
	Jacobsen 2024 (83)	HR 1.60 (0.98–1.40), as reported; point estimate lies outside the reported CI. Internally inconsistent and was not quantitatively interpreted.
Subgroup-specific	—	—
Psoriasis		
Higher Occurrence	Conrad 2023 (82)	IRR 1.3 (1.2–1.5)
	Capkun 2015 (80)	ERR 1.4 (1.2–1.5)
	Roshanisefat 2012 (39)	HR 2.27 (1.81–2.84)
	Marrie 2017 (45)	IRR 1.48 (1.09–2.00) HR 1.54 (1.07–2.24)
No Clear Difference	Nielsen 2008 (84)	RR 1.5 (0.95–2.4)
Lower Occurrence	—	—
Descriptive	—	—
Subgroup-specific	—	—
Inflammatory bowel disease		
Higher Occurrence	Karimi 2025 (85)	Crohn’s: IRR 1.69 (1.28–2.22) UC: IRR 1.42 (1.20–1.70)
	Conrad 2023 (82)	IRR 1.5 (1.2–1.8)
	Capkun 2015 (80)	UC: ERR 2.0 (1.7–2.3)
	Roshanisefat 2012 (39)	Crohn’s: HR 1.70 (1.23–2.34) UC: HR 1.81 (1.38–2.39)
No Clear Difference	—	—
Lower Occurrence	—	—
Descriptive	Pérez Díaz del Campo 2023 (53)	5/13 (38.5%)
	Kimura 2000 (86)	UC: 3/4 (75%)
Subgroup-specific	Nielsen 2008 (84)	Crohn’s: RR 0.7 (0.3–1.5) UC: RR 2.0 (1.4–2.8)
Rheumatoid arthritis		
Higher Occurrence	Kwon 2025 (87)	HR 3.32 (1.78–6.19)
	Conrad 2023 (82)	IRR 1.3 (1.1–1.5)
	Tseng 2016 (88)	HR 1.78 (1.24–2.56)
No Clear Difference	Roshanisefat 2012 (39)	HR 0.89 (0.73–1.09)
	Somers 2009 (81)	SIR 79.8 (54–114)
Lower Occurrence	Nielsen 2008 (84)	RR 0.5 (0.4–0.8)
	—	—
Descriptive	Żur-Wyrozumska 2026 (56)	225/464 (48.5%)
Subgroup-specific	—	—
Systemic lupus erythematosus		
Higher Occurrence	Kwon 2025 (87)	HR 12.25 (4.12–36.44)
	Conrad 2023 (82)	IRR 2.1 (1.6–2.9)
No Clear Difference	Nielsen 2008 (84)	RR 0.5 (0.1–2.0)
Lower Occurrence	—	—
Descriptive	Fanouriakis 2014 (58)	4/9 (44.4%)
Subgroup-specific	—	—
Vitiligo		
Higher Occurrence	—	—
No Clear Difference	—	—
Lower Occurrence	Conrad 2023 (82)	IRR 0.5 (0.3–0.9)
Descriptive	—	—
Subgroup-specific	—	—
Atopic dermatitis		
Higher Occurrence	—	—
No Clear Difference	Roshanisefat 2012 (39)	Eczema: HR 1.25 (0.49–3.21)
Lower Occurrence	—	—
Descriptive	—	—
Subgroup-specific	—	—
Celiac disease		
Higher Occurrence	—	—
No Clear Difference	Conrad 2023 (82)	IRR 1.3 (1.0–1.8)
	Ludvigsson 2007 (90)	OR 1.8 (0.9–3.7)
Lower Occurrence	—	—
Descriptive	—	—
Subgroup-specific	—	—
Bullous pemphigoid		
Higher Occurrence	Roshanisefat 2012 (39)	HR 13.50 (7.92–23.16)
	Nielsen 2008 (84)	RR 15.4 (8.7–27.1)
No Clear Difference	—	—
Lower Occurrence	—	—
Descriptive	Langer-Gould 2010 (32)	4/4
Subgroup-specific	—	—
Primary Sjögren’s syndrome		
Higher Occurrence	Kwon 2025 (87)	HR 6.16 (1.80–21.04)
	Conrad 2023 (82)	IRR 1.8 (1.3–2.5)
No Clear Difference	Nielsen 2008 (84)	RR 0.6 (0.2–2.5)
Lower Occurrence	—	—
Descriptive	—	—
Subgroup-specific	—	—
Systemic sclerosis		
Higher Occurrence	—	—
No Clear Difference	Conrad 2023 (82)	IRR 1.7 (0.9–3.1)
	Nielsen 2008 (84)	RR 1.6 (0.6–4.2)
Lower Occurrence	—	—
Descriptive	—	—
Subgroup-specific	—	—

CI, confidence interval; ERR, event rate ratio; HR, hazard ratio; IRR, incidence rate ratio; OR, odds ratio; RR, relative risk; SIR, standardized incidence ratio; UC, ulcerative colitis. Values in parentheses following effect estimates represent 95% confidence intervals unless otherwise specified.

### 5.1 Autoimmune thyroid disease

Several studies reported increased occurrence of thyroid disease after MS diagnosis. An American matched-cohort study reported a higher post-index rate of broadly defined thyroid gland disorders among PwMS (80), while a British population-based study reported a standardized incidence rate (SIR) of 144.4 (95% CI 110–185) for autoimmune thyroiditis following MS diagnosis (81). In contrast, a Canadian study reported no clear difference in incidence rate ratio (IRR) for subsequent thyroid disease (adjusted IRR 0.98, 95% CI 0.73–1.30) (35). Disease-specific findings were also heterogeneous. A British longitudinal study reported a higher adjusted IRR of 1.60 (95% CI 1.3–1.8) for Graves’ disease, whereas the estimate for Hashimoto’s thyroiditis was closer to the null (adjusted IRR 1.10, 95% CI 1.0–1.2) (82). Overall, thyroid disease showed evidence of increased occurrence after MS diagnosis in some large population-based datasets, although the magnitude of association varied by thyroid phenotype.

### 5.2 Type 1 diabetes

Evidence for T1D diagnosed following MS was limited. A Swedish population-based cohort reported a higher rate of T1D diagnosed after MS compared to matched controls (adjusted HR 1.45, 95% CI 1.34–1.57) (39). A Danish nationwide cohort reported an HR of 1.60 with a 95% CI of 0.98–1.40 (83). However, the point estimate lies outside the reported confidence interval. Because this internal inconsistency originates from the published source and could not be resolved, the result was not quantitatively interpreted or included in the directional synthesis. A large British longitudinal study observed no post-MS T1D cases during the study period and therefore did not yield an estimable comparative association (82). Given the small number of temporally ordered studies and limited interpretable comparative evidence, the association between occurrences of T1D following MS remains inadequately supported.

### 5.3 Psoriasis

Psoriasis showed relatively consistent evidence of increased occurrence after MS diagnosis. A British population-based study reported an adjusted IRR of 1.30 (95% CI 1.2–1.5) (82), while a matched-cohort study in the US found an event rate ratio (ERR) of 1.4 (1.2–1.5) (80). Similarly, a Swedish cohort reported a larger association, with an adjusted HR of 2.27 (95% CI 1.81–2.84) (39), and a Canadian study reported increased post-MS psoriasis occurrence (adjusted HR 1.54, 95% CI 1.07–2.24) (45). In contrast, a Danish nationwide study reported a positive but imprecise estimate, with an adjusted relative risk (RR) of 1.5 (95% CI 0.95–2.4) (84). Overall, psoriasis was one of the most consistently reported immune-mediated diseases that occur following an MS diagnosis.

### 5.4 Inflammatory bowel disease

IBD also showed recurrent evidence of increased occurrence following MS. A recent matched-cohort study in the US reported higher subsequent incidences of both Crohn’s disease (adjusted IRR 1.69, 95% CI 1.28–2.22) and ulcerative colitis (adjusted IRR 1.42, 95% CI 1.20–1.70) (85). A British longitudinal study reported an overall IBD IRR of 1.5 (95% CI 1.2–1.8) (82), while another American matched-cohort study specifically noted a higher event rate for ulcerative colitis (ERR 2.0, 95% CI 1.7–2.3) (80). Similarly, a Swedish population-based study reported higher rates of Crohn’s disease (adjusted HR 1.70, 95% CI 1.23–2.34) and ulcerative colitis (adjusted HR 1.81, 95% CI 1.38–2.39) after MS (39).

A Danish registry study identified subtype-specific differences, reporting no clear increase in Crohn’s disease (adjusted RR 0.7, 95% CI 0.3–1.5), but a notable rise in ulcerative colitis occurrence (adjusted RR 2.0, 95% CI 1.4–2.8) (84). Descriptive studies reported mixed temporal patterns, with IBD diagnosed after MS in 5 out of 13 overlap cases (53), and ulcerative colitis diagnosed after MS in 3 out of 4 identified overlap cases that had a known diagnostic sequence (86). In summary, the evidence most consistently supported increased subsequent ulcerative colitis, while findings for Crohn’s disease were somewhat more variable.

### 5.5 Rheumatoid arthritis

The findings on the relationship between MS and subsequent RA were diverse, with both positive and inverse associations noted. A South Korean nationwide cohort reported a higher risk of developing RA after MS (HR 3.32, 95% CI 1.78–6.19) (87). Similarly, a British longitudinal study reported an adjusted IRR of 1.3 (95% CI 1.1–1.5) (82), and a Taiwanese nationwide cohort reported an adjusted HR of 1.78 (95% CI 1.24–2.56) (88). In contrast, a Danish nationwide study reported a lower rate of subsequent RA (adjusted RR 0.5, 95% CI 0.4–0.8) (84).

On the other hand, a Swedish cohort reported no clear difference in RA risk following an MS diagnosis compared to individuals without MS, reporting an adjusted HR of 0.89 (95% CI, 0.73–1.09) (39). Likewise, another British population-based study showed no clear difference (SIR 79.8, 95% CI 54–114) (81). Descriptive data from another Polish nationwide registry study observed that 225 of 464 individuals with both conditions were diagnosed with MS prior to RA (56). Overall, available studies reported inconsistent findings on the risk of RA following MS across different populations and studies.

### 5.6 Systemic lupus erythematosus

Evidence regarding SLE occurring after MS diagnosis was limited and heterogeneous. A South Korean nationwide cohort study reported a substantially increased risk of subsequent SLE among PwMS (HR 12.25, 95% CI 4.12–36.44) (87), and a British population-based longitudinal study also observed higher SLE incidence following MS (adjusted IRR 2.1, 95% CI 1.6–2.9) (82). In contrast, a Danish nationwide study yielded a lower point estimate with substantial imprecision (adjusted RR 0.5, 95% CI 0.1–2.0) (84). Descriptive evidence from a Greek single-center cohort identified SLE developing after MS in 4 of 9 individuals with both conditions (58). These findings suggest that subsequent SLE has been observed among PwMS, but the evidence base remains small and inconsistent.

### 5.7 Vitiligo

Vitiligo was rarely examined in temporally ordered analyses. A large British longitudinal study reported a lower incidence of vitiligo following MS diagnosis (adjusted IRR 0.5, 95% CI 0.3–0.9) (82). Given that this finding was based on a single study, the evidence remains limited to determine whether MS is consistently associated with lower subsequent vitiligo occurrence.

### 5.8 Atopic dermatitis

Evidence for atopic dermatitis or eczema following MS was sparse. A Swedish cohort reported no clear difference in subsequent eczema in PwMS compared to matched controls (adjusted HR 1.25, 95% CI 0.49–3.21) (39). The limited number of studies available precludes a clear conclusion on the risk of developing atopic dermatitis or eczema after MS.

### 5.9 Celiac disease

Few studies evaluated celiac disease occurring after MS diagnosis. A British longitudinal study reported a modestly elevated incidence rate ratio (adjusted IRR 1.3, 95% CI 1.0–1.8) (82), while a Swedish population-based study observed a similarly positive but imprecise estimate (adjusted OR 1.8, 95% CI 0.9–3.7) (90). Although both studies reported point estimates above the null, the confidence intervals indicated substantial uncertainty and did not provide consistent evidence of increased celiac disease occurrence following MS.

### 5.10 Bullous pemphigoid

Bullous pemphigoid demonstrated some of the strongest associations among the immune-mediated conditions examined after MS diagnosis. A Swedish population-based cohort reported a markedly increased risk of subsequent bullous pemphigoid (adjusted HR 13.50, 95% CI 7.92–23.16) (39), while a Danish nationwide study reported a similarly elevated occurrence (adjusted RR 15.4, 95% CI 8.7–27.1) (84). An American population-based study also found that all four bullous pemphigoid cases identified among PwMS occurred after MS onset, providing additional descriptive support for a temporal sequence (32). Although these studies consistently reported large effect estimates, bullous pemphigoid remained an uncommon outcome, and the relatively small number of observed cases should be considered when interpreting the magnitude and precision of the association.

### 5.11 Primary Sjögren’s syndrome

Primary Sjögren’s syndrome showed positive associations in two large cohorts. A South Korean nationwide cohort reported an increased risk of subsequent primary Sjögren’s syndrome (HR 6.16, 95% CI 1.80–21.04) (87), while a British longitudinal study similarly observed a higher incidence following MS (adjusted IRR 1.8, 95% CI 1.3–2.5) (82). In contrast, a Danish nationwide registry study yielded a lower but highly imprecise estimate (adjusted RR 0.6, 95% CI 0.2–2.5) (84). Collectively, the available evidence suggests that primary Sjögren’s syndrome may occur more frequently among PwMS, although the number of studies remains small and findings have not been entirely consistent.

### 5.12 Systemic sclerosis

Systemic sclerosis was infrequently evaluated, and the available evidence was sparse. A British longitudinal study reported a positive but imprecise estimate for systemic sclerosis following MS (adjusted IRR 1.70, 95% CI 0.9–3.1) (82), and a Danish nationwide study observed a similarly uncertain estimate (adjusted RR 1.6, 95% CI 0.6–4.2) (84). Although both point estimates were directionally positive, the wide confidence intervals indicate substantial uncertainty. The current evidence remains insufficient to support a consistent increase in systemic sclerosis occurrence following MS.

## 6 Associations between previously diagnosed immune-mediated diseases and subsequently diagnosed MS

Studies evaluating associations between previously diagnosed immune-mediated disease and subsequently diagnosed MS reported heterogeneous findings across conditions (**Table 3**). Most studies established temporal sequence based on recorded diagnostic dates rather than the date of the first MS symptom or biological disease onset. Therefore, these findings indicate only the sequence of recorded diagnoses and do not confirm that an immune-mediated condition preceded the biological or symptomatic onset of MS, caused MS, or represented part of an MS prodrome.

**Table 3:** Associations between previously diagnosed immune-mediated diseases and subsequently diagnosed MS.

Categorization	Reference(s)	Findings
Autoimmune thyroid disease / thyroid disease		
Higher Occurrence	—	—
No Clear Difference	Langer-Gould 2010 (32)	Graves’: OR 0.7 (0.2–2.9) Hashimoto’s: OR 5.0 (0.3–80.2)
	Somers 2009 (81)	Autoimmune: SIR 111 (70–167)
Lower Occurrence	—	—
Descriptive	—	—
Subgroup-specific	Conrad 2023 (82)	Graves’: IRR 1.2 (1.0–1.5) Hashimoto’s: IRR 1.2 (1.1–1.3)
Type 1 diabetes		
Higher Occurrence	Conrad 2023 (82)	IRR 3.4 (1.6–7.0)
	Nielsen 2006 (91)	RR 3.26 (1.80–5.88)
No Clear Difference	Roshanisefat 2012 (39)	HR 1.07 (0.94–1.21)
	Langer-Gould 2010 (32)	OR 0.8 (0.3–2.4)
	Somers 2009 (81)	SIR 120.2 (67–198)
Lower Occurrence	—	—
Descriptive	—	—
Subgroup-specific	Magyari 2014 (11)	Male: OR 3.34 (1.40–7.02) Female: no difference, OR not reported
Psoriasis		
Higher Occurrence	Egeberg 2016 (8)	Mild: IRR 1.84 (1.46–2.30) Severe: IRR 2.61 (1.44–4.74)
No Clear Difference	Conrad 2023 (82)	IRR 1.2 (1.0–1.3)
	Langer-Gould 2010 (32)	OR 1.8 (0.9–3.6)
Lower Occurrence	Roshanisefat 2012 (39)	HR 0.60 (0.38–0.93)
Descriptive	—	—
Subgroup-specific	—	—
Inflammatory bowel disease		
Higher Occurrence	Yaqubi 2024 (92)	Crohn’s: HR 2.09 (1.28–3.39) UC: HR 2.35 (1.47–3.78)
	Conrad 2023 (82)	IRR 1.6 (1.3–2.0)
	Avasarala 2021 (93)	IRR 1.32 (1.03–1.71)
	Langer-Gould 2010 (32)	OR 2.7 (1.1–6.8)
No Clear Difference	Roshanisefat 2012 (39)	Crohn’s: HR 1.26 (0.93–1.70) UC: HR 1.15 (0.85–1.55)
Lower Occurrence	—	—
Descriptive	—	—
Subgroup-specific	Magyari 2014 (11)	Crohn’s / Male: OR 5.03 (1.18–16.10)
	Gupta 2005 (50)	Crohn’s: IRR 2.12 (0.94–4.50) UC: IRR 2.63 (1.29–5.15)
Rheumatoid arthritis		
Higher Occurrence	Conrad 2023 (82)	IRR 1.7 (1.4–2.0)
No Clear Difference	Dreyer 2016 (94)	SIR 0.65 (0.24–1.72)
	Langer-Gould 2010 (32)	OR 0.5 (0.1–2.0)
	Somers 2009 (81)	SIR 73.2 (39–125)
Lower Occurrence	Roshanisefat 2012 (39)	HR 0.60 (0.45–0.79)
	Batko 2024 (89)	Lower MS occurrence during the five years following RA diagnosis; IRR and 95% CI presented graphically, with the interval below 1.
Descriptive	Żur-Wyrozumska 2026 (56)	RA was diagnosed before MS in 196/464 individuals with both conditions (42.2%); no comparator-based effect estimate reported
Subgroup-specific	—	—
Systemic lupus erythematosus		
Higher Occurrence	Conrad 2023 (82)	IRR 1.8 (1.3–2.5)
	Lin 2020 (95)	Adult: HR 2.80 (1.04–7.49) Pediatric: HR 21.65 (1.29–363.4)
No Clear Difference	Langer-Gould 2010 (32)	OR 1.3 (0.4–4.0)
Lower Occurrence	—	—
Descriptive	—	—
Subgroup-specific	Magyari 2014 (11)	Male: OR 12.55 (1.62–69.95) Female: no clear difference
Vitiligo		
Higher Occurrence	—	—
No Clear Difference	Conrad 2023 (82)	IRR 1.0 (0.7–1.4)
Lower Occurrence	—	—
Descriptive	—	—
Subgroup-specific	—	—
Atopic dermatitis		
Higher Occurrence	—	—
No Clear Difference	de Lusignan 2022 (96)	HR 0.95 (0.68–1.35)
	Krishna 2019 (97)	Eczema: IRR 1.05 (0.95–1.17)
	Roshanisefat 2012 (39)	Eczema: HR 0.91 (0.56–1.48)
	Langer-Gould 2010 (32)	OR 1.1 (0.7–1.7)
	Alonso 2006 (98)	Eczema: OR 1.1 (0.6–2.1)
Lower Occurrence	—	—
Descriptive	Sahraian 2013 (99)	Eczema: 28.2% vs 27.7%, *p* = 0.91
Subgroup-specific	—	—
Asthma		
Higher Occurrence	Ponsonby 2006 (67)	OR 1.97 (95% CI 1.10–3.51)
No Clear Difference	Krishna 2019 (97)	IRR 1.07 (0.97–1.18)
	Langer-Gould 2010 (32)	OR 0.9 (0.7–1.2)
	Alonso 2006 (98)	OR 0.9 (0.4–2.0)
Lower Occurrence	Pedotti 2009 (100)	OR 0.38 (0.22–0.66)
Descriptive	Sahraian 2013 (99)	0.51% vs 4.62%, *p* = 0.02
Subgroup-specific	—	—
Celiac disease		
Higher Occurrence	—	—
No Clear Difference	Nilsson 2026 (101)	HR 1.31 (0.54–3.19)
	Conrad 2023 (82)	IRR 1.4 (1.0–1.9)
	Ludvigsson 2007 (90)	HR 1.5 (0.7–3.1)
Lower Occurrence	—	—
Descriptive	—	—
Subgroup-specific	—	—
Bullous pemphigoid		
Higher Occurrence	Kibsgaard 2017 (76)	HR 9.4 (4.9–18.0)
No Clear Difference	—	—
Lower Occurrence	—	—
Descriptive	—	—
Subgroup-specific	—	—
Primary Sjögren’s syndrome		
Higher Occurrence	Conrad 2023 (82)	IRR 1.9 (1.3–2.9)
	Goulabchand 2022 (102)	HR 3.66 (2.35–5.68)
No Clear Difference	—	—
Lower Occurrence	—	—
Descriptive	—	—
Subgroup-specific	—	—
Systemic sclerosis		
Higher Occurrence	Conrad 2023 (82)	IRR 2.3 (1.2–4.3)
	Tseng 2016 (103)	HR 69.48 (21.69–222.54)
No Clear Difference	—	—
Lower Occurrence	—	—
Descriptive	—	—
Subgroup-specific	—	—
Uveitis		
Higher Occurrence	Langer-Gould 2010 (32)	OR 3.2 (1.7–5.7)
No Clear Difference	—	—
Lower Occurrence	—	—
Descriptive	—	—
Subgroup-specific	—	—

CI, confidence interval; HR, hazard ratio; IRR, incidence rate ratio; MS, multiple sclerosis; OR, odds ratio; RA, rheumatoid arthritis; RR, relative risk; SIR, standardized incidence ratio; UC, ulcerative colitis. Values in parentheses following effect estimates represent 95% confidence intervals unless otherwise specified.

### 6.1 Autoimmune thyroid disease

Findings regarding previously diagnosed autoimmune thyroid disease and subsequently diagnosed MS varied across studies and thyroid phenotypes. An American population-based study yielded a higher but imprecise estimate (adjusted OR 5.0, 95% CI 0.3–80.2) for Hashimoto’s thyroiditis, and no clear association for Graves’ disease (adjusted OR 0.7, 95% CI 0.2–2.9) (32). A British population-based study showed no clear association for autoimmune thyroid disease, with a SIR of 111.0 (95% CI 70–167) (81). Another population-based longitudinal study from the UK reported modest disease subtype-specific estimates for Graves’ disease (adjusted IRR 1.2, 95% CI 1.0–1.5) and Hashimoto thyroiditis (adjusted IRR 1.2, 95% CI 1.1–1.3) (82). Across available studies, evidence does not demonstrate a consistent association between previously diagnosed autoimmune thyroid disease and subsequently diagnosed MS across studies and thyroid phenotypes.

### 6.2 Type 1 diabetes

Evidence regarding the association between previously diagnosed T1D and subsequently diagnosed MS was mixed. Two population-based studies reported higher subsequent MS occurrence. In a Danish cohort, 11 MS cases occurred among 6,078 individuals with T1D during 71,626 person-years of follow-up, compared with 3.38 expected cases (adjusted RR 3.26, 95% CI 1.80–5.88) (91). Similarly, a British population-based longitudinal study reported an adjusted IRR of 3.4 (95% CI 1.6–7.0) (82).

In contrast, several other studies did not find a clear association. Reported estimates included an adjusted HR of 1.07 (95% CI 0.94–1.21) (39), an adjusted OR of 0.8 (95% CI 0.3–2.4) (32), and an SIR of 120.2 (95% CI 67–198) (81). One study also reported sex-specific findings, with higher odds of prior T1D diagnosis among men with MS (OR 3.34, 95% CI 1.40–7.02), while no corresponding difference was observed among women (11). Overall, some studies indicated a higher occurrence of subsequently diagnosed MS among individuals with previously diagnosed T1D, but the association was not consistent across studies and may vary by sex.

### 6.3 Psoriasis

Several studies examined the association between previously diagnosed psoriasis and subsequently diagnosed MS, with mixed findings. A large Danish nationwide cohort reported higher MS incidence among individuals with both mild psoriasis (adjusted IRR 1.84, 95% CI 1.46–2.30) and severe psoriasis (adjusted IRR 2.61, 95% CI 1.44–4.74) compared with general population without psoriasis, with a larger estimate observed for severe disease (8). Other studies provided less clear evidence of higher subsequent MS occurrence. A longitudinal study from the UK reported a modest estimate whose confidence interval reached the null value (adjusted IRR 1.2, 95% CI 1.0–1.3) (82), while an American population-based study reported a numerically increased but imprecise association (adjusted OR 1.8, 95% CI 0.9–3.6) (32). Conversely, a Swedish population-based study reported lower occurrence of prior psoriasis diagnosis among PwMS (adjusted HR 0.60, 95% CI 0.38–0.93) (39). Overall, one large cohort supported higher subsequent MS occurrence among individuals with psoriasis, whereas other studies reported uncertain or inverse associations, resulting in an inconsistent overall pattern.

### 6.4 Inflammatory bowel disease

Several studies reported higher occurrence of subsequently diagnosed MS among individuals with previously diagnosed IBD. In a large cohort in Germany, 10-year MS incidence was higher among both patients with Crohn’s disease (0.9% vs 0.5%; HR 2.09, 95% CI 1.28–3.39) and ulcerative colitis (0.7% vs 0.3%; HR 2.35, 95% CI 1.47–3.78) than among matched non-IBD controls (92). Additionally, other studies reported higher estimates for IBD overall, including a British cohort reporting an adjusted IRR of 1.6 (95% CI 1.3–2.0) (82), an American cohort study that found a higher subsequent incidence of MS among individuals with IBD than matched non-IBD controls (IRR 1.32, 95% CI 1.03–1.71, *p* = 0.03) (93), and an American case-control study that reported an increased association between IBD and MS (adjusted OR 2.7, 95% CI 1.1–6.8, *p* = 0.03) (32).

However, findings were not uniform across studies or IBD subtypes. A Swedish population-based study found no clear association for either Crohn’s disease (adjusted HR 1.26, 95% CI 0.93–1.70) or ulcerative colitis (adjusted HR 1.15, 95% CI 0.85–1.55) (39). Subgroup-specific findings were also reported. One study from Denmark observed a higher but imprecise estimate for Crohn’s disease among men (OR 5.03, 95% CI 1.18–16.10) (11), while another British study reported a positive but imprecise estimate for Crohn’s disease (IRR 2.12, 95% CI 0.94–4.50) alongside a higher estimate for ulcerative colitis (IRR 2.63, 95% CI 1.29–5.15) (50). Taken together, the available evidence suggests that previously diagnosed IBD may be associated with a higher occurrence of subsequently diagnosed MS, although the magnitude and consistency of the association varied by study, IBD subtype, and subgroup.

### 6.5 Rheumatoid arthritis

Studies examining the association between previously diagnosed RA and subsequently diagnosed MS were heterogeneous. A British longitudinal study reported a higher occurrence of subsequently diagnosed MS following RA diagnosis (adjusted IRR 1.7, 95% CI 1.4–2.0) (82). In contrast, several other studies found no clear association. In an American population-based study, prior RA was identified in 0.2% of PwMS compared with 0.4% of controls (adjusted OR 0.5, 95% CI 0.1–2.0) (32). Similarly, a British population-based study identified 13 subsequent MS cases among individuals with RA (SIR 73.2, 95% CI 39–125) (81), while a Danish nationwide cohort identified 12 incident MS cases following RA diagnosis (SIR 0.65, 95% CI 0.24–1.72) (94). Conversely, a Swedish population-based study reported a lower risk of subsequent MS among individuals with RA (adjusted HR 0.60, 95% CI 0.45–0.79) (39). A Polish RA-indexed matched case-control study also reported a lower MS occurrence in the five years following an RA diagnosis than in matched population controls. The IRR was presented graphically and remained below 1, with a 95% CI excluding the null; however, the exact numerical values were not reported (89). A Polish population-based study reported that RA was diagnosed before MS in 196 of 464 individuals with both conditions (42.2%); however, because the study did not provide a comparator-based effect estimate for this diagnostic sequence, the finding was considered descriptive and does not establish a higher or lower occurrence of subsequently diagnosed MS (56). Overall, the evidence does not demonstrate a consistent association between previously diagnosed RA and subsequently diagnosed MS, with higher, imprecise, and inverse estimates reported across studies.

### 6.6 Systemic lupus erythematosus

SLE was evaluated in a limited number of studies examining occurrence of subsequently diagnosed MS. A British population-based longitudinal study reported a higher occurrence of subsequently diagnosed MS among individuals with SLE (adjusted IRR 1.8, 95% CI 1.3–2.5) (82). Additional evidence came from more selected populations. In a nationwide Taiwanese study of individuals with optic neuritis, SLE was associated with a higher risk of subsequent MS, both in adults (adjusted HR 2.80; 95% CI 1.04–7.49, *p* = 0.04) and pediatric patients (adjusted HR 21.65, 95% CI 1.29–363.4, *p* = 0.03) (95). However, the pediatric estimate was highly imprecise. An American population-based study found no clear association (adjusted OR 1.3, 95% CI 0.4–4.0) (32). Another study reported a sex-specific association among men (OR 12.55, 95% CI 1.62–69.95), but the estimate was highly imprecise, and no clear difference was observed among women (11). Collectively, although certain studies reported higher estimates, the limited number of available studies, reliance on population-specific analyses, and imprecision in several estimates prevent a definitive conclusion regarding the association between previously diagnosed SLE and subsequently diagnosed MS.

### 6.7 Vitiligo

Evidence regarding the association between previously diagnosed vitiligo and subsequently diagnosed MS was limited to one study. This study reported no clear difference in the occurrence of subsequently diagnosed MS between individuals with vitiligo and the comparator population (adjusted IRR 1.0, 95% CI 0.7–1.4) (82). Therefore, the available evidence is insufficient to establish a consistent association between previously diagnosed vitiligo and subsequently diagnosed MS.

### 6.8 Atopic dermatitis

Studies examining the association between previously diagnosed atopic dermatitis or eczema and subsequently diagnosed MS generally found no clear difference. Reported values of adjusted estimates included an HR of 0.95 (95% CI 0.68–1.35) (96), an IRR of 1.05 (95% CI 0.95–1.17) (97), an HR of 0.91 (95% CI 0.56–1.48) (39), an OR of 1.1 (95% CI 0.7–1.7) (32), and an OR of 1.1 (95% CI 0.6–2.1) (98). One additional study examining an Iranian cohort reported similar frequencies of eczema among individuals with and without MS (28.2% vs 27.7%, *p* = 0.91) but did not provide a comparative effect estimate (99). Overall, the available evidence does not demonstrate a consistent association between previously diagnosed atopic dermatitis and subsequently diagnosed MS.

### 6.9 Asthma

Findings regarding previously diagnosed asthma and subsequently diagnosed MS were heterogeneous, with higher, near-null, and lower estimates reported across studies. An Australian population-based case-control study reported higher odds of asthma onset before recorded MS diagnosis among PwMS than among controls (OR 1.97, 95% CI 1.10–3.51) (67). In contrast, an Italian population-based case-control study reported lower odds of asthma or allergic asthma among PwMS than among controls (OR 0.38; 95% CI 0.22–0.66; *p* < 0.01) (100). Additionally, another study from Iran reported a lower frequency of pre-MS asthma among PwMS compared to controls (0.51% versus 4.62%, *p* = 0.02) but did not provide a comparative effect estimate (99). Some additional studies also examined the occurrence of subsequent MS after the diagnosis of asthma but reported a near-null effect estimate and no clear differences (32, 97, 98). Overall, the available evidence does not demonstrate a consistent direction of association between previously diagnosed asthma and subsequently diagnosed MS.

### 6.10 Other immune-mediated diseases diagnosed before subsequent MS

Several additional immune-mediated diseases were examined in relation to subsequently diagnosed MS. Three studies found no clear association between previously diagnosed celiac disease and subsequently diagnosed MS, with adjusted estimates of HR 1.31 (95% CI 0.54–3.19) (101), IRR 1.4 (95% CI 1.0–1.9) (82), and HR 1.5 (95% CI 0.7–3.1) (90), from population-based studies conducted in Finland, UK, and Sweden, respectively.

In a Danish population-based study, bullous pemphigoid was associated with a higher occurrence of subsequent MS during follow-up (HR 9.4, 95% CI 4.9–18.0), even when events during the first year after diagnosis of bullous pemphigoid were excluded (HR 5.1, 95% CI 2.3–11.3) (76). Although this establishes the sequence of recorded diagnoses, it does not demonstrate that bullous pemphigoid preceded the biological or symptomatic onset of MS or represented an MS prodrome.

For primary Sjögren’s syndrome, one study reported a higher occurrence of subsequently diagnosed MS (adjusted IRR 1.9, 95% CI 1.3–2.9) (82), while a French cohort reported increased subsequent hospitalization for MS (adjusted HR 3.66, 95% CI 2.35–5.68, *p* < 0.001) (102). The latter outcome reflects MS-related hospitalization rather than necessarily a new MS diagnosis.

Systemic sclerosis was also associated with subsequently diagnosed MS. One longitudinal study conducted in the UK reported an adjusted IRR of 2.3 (95% CI 1.2–4.3) (82). A Taiwanese cohort identified 54 incident cases of MS among individuals with systemic sclerosis during a follow-up period of 7.45 ± 3.72 years (adjusted HR 69.48, 95% CI 21.69–222.54) (103).

Previously diagnosed uveitis was also associated with subsequently diagnosed MS in one population-based study (adjusted OR 3.2, 95% CI 1.7–5.7) (32).

Overall, these findings varied substantially across conditions and studies. As several estimates were based on uncommon outcomes or specific populations, these findings should be interpreted with caution and do not establish a generalized association between immune-mediated diseases and subsequent diagnosis of MS.

## 7 Impact of immune-mediated comorbidities on MS disease course

Studies evaluating associations between immune-mediated comorbidities and MS disease course yielded heterogeneous but clinically relevant findings (**Table 4**). Outcomes of interest included MRI measures of focal inflammatory activity or brain damage, relapses, and disability progression among PwMS with and without immune-mediated comorbidities.

**Table 4:** Associations between immune-mediated comorbidities and MS clinical outcomes.

Categorization	Reference(s)	Findings
Autoimmune thyroid disease / thyroid disease		
Worse Outcomes	Zivadinov 2016 (13)	Lower normalized brain volume (1454.3 vs 1481.0 mL); gray-matter volume (722.9 vs 747.9 mL); cortical volume (537.9 vs 557.6 mL); all corrected *p* < 0.05
No Clear Difference	Kaya 2025 (104)	Five-year median EDSS: 1.25 vs 1.25; new MRI lesions: 10 vs 9; ≥1 relapse: 9 vs 11; NEDA-3: 13 vs 12; all *p* > 0.05
	Lorefice 2018 (105)	Gray-matter volume: β = 9.0 (95% CI −5.3 to 23.3); cortical gray-matter volume: β = 11.1 (95% CI −0.7 to 22.9)
Better Outcomes	—	—
Descriptive	—	—
Subgroup-specific	—	—
Type 1 diabetes		
Worse Outcomes	Binzer 2024 (106)	EDSS 6: HR 2.21 (95% CI 1.48–3.31); EDSS 3: HR 1.25 (95% CI 0.91–1.72); EDSS 4: HR 1.38 (95% CI 0.93–2.04); higher risk of sustained EDSS 6
	Lorefice 2018 (105)	Gray-matter volume: β = −18.6 (95% CI −36.1 to −1.0); cortical gray-matter volume: β = −15.5 (95% CI −30.1 to −1.0)
No Clear Difference	—	—
Better Outcomes	—	—
Descriptive	—	—
Subgroup-specific	—	—
Psoriasis		
Worse Outcomes	Zivadinov 2016 (13)	Lower normalized brain volume (1394.3 vs 1481.0 mL, corrected *p* < 0.001); gray-matter and cortical volumes (694.4 vs 747.9 and 516.2 vs 557.6 mL, both *p* < 0.01); white-matter volume and normal-appearing brain-tissue MTR (699.8 vs 732.6 mL and 33.1 vs 35.3, both *p* < 0.05)
No Clear Difference	Brummer 2026 (108)	Mean EDSS: 2.53 vs 2.08, adjusted *p* = 0.389
	Tettey 2016 (42)	EDSS: β = −0.03 (95% CI −0.85 to 0.79); MSSS: β = 0.19 (95% CI −1.11 to 1.49); relapse: HR 0.50 (95% CI 0.18–1.42)
Better Outcomes	—	—
Descriptive	—	—
Subgroup-specific	Miron 2017 (107)	2^nd^ relapse: HR 0.56, *p* = 0.01; EDSS 4: HR 0.31, *p* = 0.03; EDSS 6: HR 0.18, *p* = 0.03; psoriasis before MS was associated with longer time to 2^nd^ relapse and EDSS 4/6; psoriasis after MS did not differ
Inflammatory bowel disease		
Worse Outcomes	Binzer 2024 (106)	Crohn’s disease: EDSS 3: HR 2.30 (95% CI 1.74–3.04); EDSS 4: HR 1.59 (95% CI 1.09–2.32); EDSS 6: HR 1.61 (95% CI 0.98–2.64) Ulcerative colitis: EDSS 3: HR 1.57 (95% CI 1.15–2.14); EDSS 4: HR 0.97 (95% CI 0.60–1.56); EDSS 6: HR 1.29 (95% CI 0.73–2.27)
No Clear Difference	—	—
Better Outcomes	Zéphir 2014 (109)	EDSS 3: OR 0.45 (95% CI 0.24–0.88); EDSS 4: OR 0.48 (95% CI 0.20–0.90); SPMS conversion: OR 0.44 (95% CI 0.20–0.98); relapse rate: OR 1.10 (95% CI 0.49–2.42)
Descriptive	—	—
Subgroup-specific	—	—
Rheumatoid arthritis		
Worse Outcomes	Tettey 2016 (42)	Relapse: HR 3.70 (95% CI 1.80–7.58); EDSS: β = 0.71 (95% CI −0.78 to 2.19); MSSS: β = 0.69 (95% CI −1.63 to 3.00)
No Clear Difference	Zivadinov 2016 (13)	No clear differences in primary MRI outcomes after correction; normalized brain volume: 1461.2 vs 1481.0 mL; T2 lesion volume: 17.1 vs 15.3 mL; normal-appearing brain-tissue MTR: 33.0 vs 35.3; all corrected *p* > 0.05
Better Outcomes	—	—
Descriptive	—	—
Subgroup-specific	—	—
Systemic lupus erythematosus		
Worse Outcomes	—	—
No Clear Difference	—	—
Better Outcomes	—	—
Descriptive	Nikolopoulos 2022 (110)	Relapse: OR 18.2 (95% CI 1.76–188); comparator was SLE-related demyelination rather than PwMS without SLE
	Fanouriakis 2014 (58)	During a median four-year follow-up, 3/9 patients remained neurologically stable and 6/9 experienced gradual deterioration; SLE remained quiescent in all patients; no comparative estimate of the effect of SLE on MS outcomes
Subgroup-specific	—	—
Atopic dermatitis		
Worse Outcomes	—	—
No Clear Difference	Tettey 2016 (42)	EDSS: β = 0.16 (95% CI −0.47 to 0.78); MSSS: β = 0.38 (95% CI −0.61 to 1.37); relapse: HR 1.26 (95% CI 0.74–2.16)
Better Outcomes	—	—
Descriptive	—	—
Subgroup-specific	—	—
Asthma		
Worse Outcomes	—	—
No Clear Difference	Tettey 2016 (42)	EDSS: β = 0.02 (95% CI −0.51 to 0.56); MSSS: β = 0.04 (95% CI −0.76 to 0.87); relapse: HR 1.09 (95% CI 0.27–1.44)
	Zivadinov 2016 (13)	No clear differences in primary MRI outcomes after correction; normalized brain volume: 1490.6 vs 1481.0 mL; T2 lesion volume: 12.9 vs 15.3 mL; normal-appearing brain-tissue MTR: 36.4 vs 35.3; all corrected *p* > 0.05
	Bourne 2017 (65)	Annualized relapse rate: 0.38 vs 0.40, *p* = 0.85.
	Manouchehrinia 2015 (66)	EDSS 4: HR 1.29 (95% CI 0.93–1.77); EDSS 6: HR 1.33 (95% CI 0.93–1.89)
Better Outcomes	—	—
Descriptive	—	—
Subgroup-specific	—	—
Celiac disease		
Worse Outcomes	—	—
No Clear Difference	—	—
Better Outcomes	—	—
Descriptive	Lorefice 2018 (105)	No clear evidence of worsening MRI measures; disease-specific quantitative comparative estimate not reported
Subgroup-specific	—	—
Uveitis		
Worse Outcomes	Martin 2023 (111)	Reached EDSS 3: 36.4% vs 18.0%, *p* = 0.047; time to EDSS 3: *p* = 0.003; faster time to EDSS 3 and more patients reaching EDSS 3
No Clear Difference	—	—
Better Outcomes	Shugaiv 2015 (112)	Final EDSS: 2.0 vs 3.0, *p* = 0.004; progression index: 0.2 vs 0.4, *p* < 0.001; progressive MS: 0% vs 36%, *p* < 0.001
Descriptive	—	—
Subgroup-specific	—	—

CI, confidence interval; EDSS, Expanded Disability Status Scale; HR, hazard ratio; MRI, magnetic resonance imaging; MS, multiple sclerosis; MSSS, Multiple Sclerosis Severity Score; MTR, magnetization transfer ratio; OR, odds ratio; PwMS, people with multiple sclerosis; SLE, systemic lupus erythematosus; SPMS, secondary progressive multiple sclerosis. β indicates the regression coefficient.

### 7.1 Autoimmune thyroid disease

Three studies evaluated associations between thyroid disease and MS outcomes. An American study reported that 97 PwMS with thyroid disease examined had significantly lower normalized brain, gray matter, and cortical volumes compared with PwMS without thyroid disease, suggesting greater brain atrophy in the comorbid group (all *p* < 0.05) (13). In contrast, one single-center study from Türkiye found no clear differences in relapse activity, MRI measures, Expanded Disability Status Scale (EDSS), or NEDA-3 status during at least 5 years of follow-up (104), while an Italian case-control study found no clear differences in brain and cortical gray-matter volumes between PwMS with and without thyroid disease (105). Overall, although thyroid disease may be clinically relevant in selected MS cohorts, the available evidence remains mixed and does not establish a consistent association with relapse activity, disability progression, or other measures of MS disease course.

### 7.2 Type 1 diabetes

Evidence regarding the association between T1D and MS outcomes was limited to two studies. In a Swedish nationwide study, PwMS with T1D had a higher risk of disability progression to EDSS 6.0 compared with PwMS without T1D (adjusted HR 2.21, 95% CI 1.48–3.31) (106). In addition, an Italian case-control MRI study reported significantly lower gray-matter and cortical gray-matter volumes among PwMS with T1D after adjustment for demographic and MS-related clinical characteristics (*p* = 0.038 and 0.036, respectively) (105). Together, these findings suggest a possible association between T1D and worse disability and MRI outcomes; however, the small number of observational studies precludes a firm conclusion and warrants confirmation in larger longitudinal cohorts.

### 7.3 Psoriasis

Psoriasis showed mixed evidence regarding the MS disease course. An American observational study reported worse MRI outcomes among PwMS with psoriasis, particularly in significantly lower normalized brain volume (*p* < 0.001), gray matter and cortical volume (both *p* < 0.01), and white matter volume (*p* < 0.05) compared with PwMS without psoriasis (13). Conversely, a case-control study assessing the effects of psoriasis onset preceding relapsing-remitting MS showed a slower disease progression in those with psoriasis, with a longer time to second relapse (HR 0.56, *p* = 0.01) and longer time to progress to EDSS 4.0 and 6.0 (HR 0.31, *p* = 0.03; HR 0.18, *p* = 0.03, respectively). The same study found no clear differences in these outcome measures when psoriasis was diagnosed after MS (107). A more recent multicenter health record review of a German cohort also did not find a significant difference in EDSS between PwMS with and without psoriasis (*p* = 0.39) (108). Similarly, an Australian population-based study found no clear association between comorbid psoriasis and disability level or relapse rate (42). Overall, although psoriasis was associated with adverse MRI measures in one study, the available evidence does not demonstrate a consistent association with MS relapse or disability progression.

### 7.4 Inflammatory bowel disease

Evidence regarding the association between IBD and MS outcomes was limited and mixed. In a Swedish cohort, Crohn’s disease was associated with a significantly higher risk of reaching EDSS 3.0 and 4.0, while ulcerative colitis was associated with increased risk of reaching EDSS 3.0 (106). However, when Crohn’s disease and ulcerative colitis were analyzed together as IBD, no clear association with reaching these EDSS thresholds was observed. Progression measured by the Normalized Age-Related Multiple Sclerosis Severity score also did not differ significantly between the periods before versus after recorded IBD diagnosis. Overall, this study provided evidence of worse disability outcomes for specific IBD subtypes, although associations were not observed consistently across all analyses.

In contrast, a French matched-cohort study reported a milder MS course among patients with concomitant IBD compared to matched PwMS without IBD, including lower median EDSS (2.5 vs 3.0; adjusted OR 0.77, 95% CI 0.64–0.92), lower odds of reaching EDSS 3.0 and 4.0, and lower odds of conversion from relapsing-remitting MS to secondary progressive MS (22% vs 34%; adjusted OR 0.44, 95% CI 0.20–0.98). Annualized relapse rates were 0.17 and 0.24, respectively, with no clear comparative difference (OR 1.10, 95% CI 0.49–2.42) (109). Given the limited and directionally heterogeneous evidence, no consistent association between IBD and MS disease course can be established.

### 7.5 Rheumatoid arthritis

Evidence regarding the impact of RA on MS outcomes was limited. An Australian population-based study found that RA comorbidity was associated with an increased risk of subsequent MS relapse (HR 3.70, 95% CI 1.80–7.58, *p* < 0.001), but no clear association with disability measured by EDSS (*p* = 0.35) (42). A cross-sectional US MRI study also found no clear differences in primary MRI outcomes between PwMS with RA and PwMS without any of the studied comorbidities after correction for multiple comparisons. Normalized brain volume was 1461.2 versus 1481.0 mL, T2 lesion volume was 17.1 versus 15.3 mL, and normal-appearing brain-tissue magnetization-transfer ratio was 33.0 versus 35.3, respectively (all corrected *p* > 0.05) (13). Overall, the limited evidence suggests a possible association between RA and relapse activity but does not demonstrate a consistent association with disability or MRI measures of brain injury.

### 7.6 Systemic lupus erythematosus

The current evidence regarding the association between comorbid SLE and MS outcomes remains insufficient. A Greek single-center study followed nine patients with coexisting SLE and MS for a median of four years. Three patients remained neurologically stable, while six experienced gradual neurological deterioration; SLE remained quiescent in all nine patients during MS treatment. Although the investigators concluded that coexistence was not associated with a severe phenotype for either disease, the study did not include a comparison with PwMS without SLE or provide a disease-specific effect estimate (58). Another Greek study reported a higher relapse burden among patients with coexisting SLE and MS compared to those with SLE-related demyelinating syndromes (OR 18.2, 95% CI 1.76–188); however, this comparator did not allow for evaluation of whether comorbid SLE is associated with worse outcomes among PwMS (110). Therefore, the available evidence is insufficient to determine whether comorbid SLE is associated with MS relapse activity, disability progression, or other clinical outcomes.

### 7.7 Other immune-mediated comorbidities

Evidence regarding other immune-mediated comorbidities and MS outcomes was limited. Atopic dermatitis was evaluated in an Australian population-based study, which reported no apparent association with MS disability measured by EDSS and relapse rate (42). Findings for asthma similarly provided no clear evidence of worse MS outcomes. A British survey study found no clear association between asthma and the likelihood of reaching EDSS 4.0 or 6.0 or with the physical component of the Multiple Sclerosis Impact Scale 29 (MSIS-29). The study reported a higher psychological MSIS-29 score among PwMS with asthma (coefficient 4.57, 95% CI 1.23–7.90, *p* = 0.007); however, this patient-reported psychological measure was not interpreted as evidence of greater MS disability progression or MRI injury (66). A pediatric multicenter study also reported similar annualized relapse rates among PwMS with and without asthma (0.38 vs 0.40, *p* = 0.85) (65). Similarly, other studies did not identify a consistent association or statistical significance between asthma and worsening of MS outcomes (13, 42).

An Italian case-control study reported no clear evidence of worse brain MRI outcomes among PwMS with celiac disease, although limited disease-specific reporting precluded a definitive conclusion (105). Uveitis/iridocyclitis showed conflicting associations with MS outcomes. A French hospital-based study reported a greater proportion of patients with comorbid uveitis reached EDSS 3.0 in a shorter duration compared to PwMS without uveitis (111), while a Turkish clinic chart review found a lower median EDSS and slower disease progression in PwMS with uveitis compared to those without (112). Overall, the prognostic impact of immune-mediated comorbidities appeared disease-specific and heterogeneous. Given the limited number of observational studies and their opposing findings, no firm conclusion can be drawn regarding the association between uveitis and MS progression. Limited evidence suggested possible adverse outcomes and MRI outcomes associated with T1D, whereas findings for thyroid disease, psoriasis, IBD, RA, and uveitis were mixed or outcome-specific. Evidence concerning SLE, atopic dermatitis, asthma, and celiac disease was sparse or inconclusive.

## 8 Discussion

A principal finding of this review is that reported associations with MS varied across immune-mediated diseases rather than demonstrating a generalized increase across all conditions. T1D and MS show evidence of recurrent epidemiological associations. Research has shown a five-fold higher prevalence of T1D in PwMS from Sardinia (40), and a three-fold higher occurrence of subsequently diagnosed MS among individuals with T1D in a Danish cohort (91). Psoriasis and IBD also show recurrent associations with MS, although the magnitude and consistency of these findings varied across populations and study designs. Mechanistic literature beyond the scope of this review has proposed potential explanations, including overlapping genetic susceptibility, impaired immune tolerance, dysregulated T- and B-cell responses, and systemic inflammatory processes (113). However, these mechanisms were not systematically evaluated in this review and should be considered as hypotheses for future research. The observed epidemiological associations may also reflect surveillance bias, diagnostic delay, treatment exposure, residual confounding, or coincidental coexistence, and therefore do not provide evidence of shared genetic or immunological pathways.

Studies investigating immune-mediated diseases diagnosed following MS were analyzed separately from co-occurrence studies. An increased subsequent incidence of psoriasis, IBD, and bullous pemphigoid was observed in several populations, while results for other conditions were less consistent. These post-MS diagnoses may be affected by treatment exposure, increased healthcare utilization, and surveillance bias, and therefore should not be considered equivalent to cross-sectional comorbidity prevalence.

Several studies also examined associations between previously diagnosed immune-mediated diseases and subsequently diagnosed MS. T1D was associated with a higher rate of subsequently diagnosed MS in a Danish cohort, with 11 observed MS cases compared to 3.38 expected cases (RR 3.26) (91). Psoriasis was also associated with subsequently diagnosed MS in a severity-dependent pattern, with incidence rates of 1.78, 3.22, and 4.55 per 10,000 person-years in the reference, mild psoriasis, and severe psoriasis populations, respectively (8). Similarly, the 10-year incidence of subsequently diagnosed MS was higher among patients with Crohn’s disease and ulcerative colitis than among matched controls (92). However, these findings were not consistent across all studies or conditions. While these studies clarify the sequence of recorded diagnoses, they do not establish whether the immune-mediated disease preceded the biological or symptomatic onset of MS. The observed associations may reflect shared genetic, environmental, or immunological susceptibility, but could also be explained by surveillance bias, diagnostic delay, treatment exposure, or residual confounding. Therefore, they should not be interpreted as evidence that these conditions represent an MS prodrome or cause MS.

Evidence concerning associations between immune-mediated comorbidities and the MS disease course was limited and heterogeneous. A small number of studies suggested possible associations between T1D and worse outcomes, but the evidence was insufficient to establish a consistent prognostic effect (105, 106). Findings for IBD, psoriasis, RA, thyroid disease, and uveitis were mixed or similarly limited. Asthma and celiac disease were not consistently associated with worse outcomes (66, 105), and SLE and atopic dermatitis had insufficient disease-specific evidence (58, 111). This pattern suggests that the presence of autoimmune comorbidity alone is not enough to predict a worse MS course. Instead, specific comorbidity types may be more clinically meaningful, especially those associated with systemic inflammatory burden, metabolic injury, or chronic immune activation.

Co-occurring immune-mediated diseases may be relevant to treatment selection, but therapeutic overlap among diseases should not be regarded as evidence of a shared pathogenesis. Sphingosine-1-phosphate receptor modulators, such as ozanimod, are approved for the treatment of MS and have demonstrated effects on lymphocyte trafficking that are relevant to other immune-mediated diseases, such as ulcerative colitis (114, 115). Similarly, anti-integrin therapies, such as natalizumab and vedolizumab, target leukocyte migration and are used in MS and IBD, respectively (116, 117). Additionally, biologic agents targeting cytokine pathways, including interleukin-17 and tumor necrosis factor (TNF) signaling, are widely used in psoriasis and other immune-mediated conditions, further supporting therapeutic overlap across several immune-mediated diseases (118, 119). These examples demonstrate that immune-directed therapies can be applied across various diseases; however, they do not provide evidence that the epidemiological associations identified in this review result from shared immunopathogenic pathways. This review did not systematically assess comparative treatment efficacy across these conditions.

Treatment exposure is also important when interpreting immune-mediated conditions diagnosed after MS. For example, Alemtuzumab, a potent anti-CD52 monoclonal antibody, is an established treatment for PwMS due to its demonstrated efficacy. However, approximately 50% of patients develop secondary autoimmune thyroid diseases, collectively termed alemtuzumab-induced autoimmune thyroid diseases, which include Graves’ disease, Hashimoto thyroiditis, and, less commonly, fluctuating thyroid dysfunction. Data from the CARE-MS studies indicate that thyroid events occurred in 40% to 46% of alemtuzumab-treated patients over six years, with most cases arising during immune reconstitution and peaking approximately three years after treatment initiation. Less frequent autoimmune complications include immune thrombocytopenia, autoimmune nephropathies, cytopenias, and autoimmune hepatitis (120, 121). Secondary autoimmune diseases have also been reported following autologous hematopoietic stem cell transplantation performed for autoimmune conditions, with frequencies ranging from approximately 2% to 14%. The incidence varies depending on the conditioning regimen, and the inclusion of alemtuzumab has been identified as a significant risk factor. An imbalance between B-cell and T-cell reconstitution is proposed as a potential mechanism underlying secondary autoimmunity after both alemtuzumab treatment and AHSCT (122). These findings indicate that treatment exposure should be considered when interpreting immune-mediated diseases diagnosed following MS and reinforce the need to distinguish treatment-associated events from underlying epidemiological co-occurrence. Several additional sources of confounding and bias should be considered when interpreting the observed associations. Age and sex influence the occurrence of both MS and many immune-mediated diseases. Geographic differences in genetic background, environmental exposures, disease prevalence, healthcare systems, and diagnostic practices may also contribute to heterogeneity across studies. Patterns of healthcare utilization can affect case ascertainment, as PwMS have been shown to use physician services more frequently than the general population, including in the years preceding MS diagnosis, thereby increasing opportunities for detecting additional conditions and introducing surveillance bias (35). Diagnostic overlap and misclassification are additional concerns, especially when neurological or systemic manifestations could be attributed to either MS or a coexisting immune-mediated disease. Studies based on administrative databases may be affected by imprecise clinical coding, variability in case definitions and coding practices, and limited clinical information necessary for diagnosis validation (123). Moreover, therapies prescribed for other immune-mediated diseases may influence the subsequent diagnosis or recognition of MS. For instance, in a retrospective study, anti-TNF-α exposure in the overall IBD population showed a higher but imprecise adjusted IRR of 1.43 (95% CI 0.62–3.32) for subsequently diagnosed MS compared to those with no exposure. The Crohn’s disease-specific estimate was similarly higher but imprecise (adjusted IRR 2.24, 95% CI 0.85–5.94), as was the estimate for ulcerative colitis (adjusted IRR 0.57, 95% CI 0.08–4.15). These estimates did not provide clear evidence of a treatment-specific association (93). Because these factors were inconsistently addressed or adjusted for across studies, residual confounding remains a significant limitation. Therefore, the observed associations should not be interpreted as causal.

Several studies reported that selected immune-mediated diseases were diagnosed before a subsequent MS diagnosis. However, this recorded diagnostic sequence does not establish that these conditions preceded the biological or symptomatic onset of MS or identify populations appropriate for early intervention. Future longitudinal studies integrating verified symptom chronology, genetic susceptibility, imaging, and biomarkers could determine whether these associations have value for risk prediction and whether biomarkers can detect neuroaxonal injury before clinical diagnosis (8, 9, 124, 125). Current evidence does not support targeted MS screening, closer neurological monitoring, preventive intervention, or preventive trials in individuals with immune-mediated diseases.

The present findings also warrant further investigation of the factors underlying the observed associations. Future research should determine whether the recurrent co-occurrence of T1D, psoriasis, and IBD with MS reflects shared genetic or immunological susceptibility; because mechanistic evidence was not systematically reviewed, these explanations remain hypotheses. Comorbid IBD or psoriasis may nevertheless be relevant to treatment selection because some immune-directed therapies are used across these diseases, while other treatments for systemic immune-mediated conditions may be inappropriate or require caution in people with demyelinating disease. Prospective studies are needed to determine the clinical and prognostic relevance of these associations and their implications for future trial design.

### 8.1 Limitations

Several limitations should be considered. First, the included studies were heterogeneous in design, population, comparator selection, and disease definitions, limiting direct comparison and preventing quantitative synthesis. Second, many studies reported relative risks, incidence rates, or observed/expected ratios rather than direct MS-versus-general-population prevalence, making it difficult to compare associations across diseases. Third, some comorbidities had sparse evidence, including Sjögren’s syndrome, systemic sclerosis, bullous pemphigoid, uveitis, and celiac disease, reducing confidence in the observed associations. Fourth, broad disease labels such as “thyroid disease,” “asthma,” or “IBD” often encompass biologically heterogeneous phenotypes, potentially obscuring disease-specific relationships with MS. Fifth, studies evaluating temporal relationships frequently used recorded diagnostic dates rather than the date of initial neurological symptoms or biological disease onset. As a result, an immune-mediated disease diagnosed prior to MS may not have preceded the actual onset of MS. These associations may be affected by surveillance bias, diagnostic delay, treatment exposure, and residual confounding, and thus cannot confirm that the preceding conditions represent an MS prodrome or play a causal role in MS development. Sixth, the studies demonstrated substantial variability in demographic, geographic, healthcare-related, diagnostic, and treatment-related factors. Incomplete adjustment for these variables, combined with potential inaccuracies in administrative case ascertainment, may have affected the reported associations. Consequently, residual confounding and ascertainment bias limit causal interpretation of the findings. Seventh, the electronic database searches were restricted to studies published from 2001 onward, whereas earlier studies were identified only through backward citation searching. Eligible studies published before 2001 may therefore have been missed. In addition, although bullous pemphigoid, primary Sjögren’s syndrome, systemic sclerosis, and uveitis were identified through the general “autoimmune disease” search term and backward reference-list screening, these conditions were not individually named in the electronic search strategy. Their ascertainment may consequently have been less comprehensive than that of conditions explicitly represented in the search terms, and the possibility of selective ascertainment cannot be excluded. This review should therefore not be interpreted as an exhaustive assessment of every possible immune-mediated comorbidity. Finally, this review was designed to synthesize epidemiological and clinical associations and did not systematically identify mechanistic, genetic, molecular, or experimental studies. As a result, the observed co-occurrence of immune-mediated diseases does not establish shared immunopathogenic pathways, biologically distinct MS subgroups, or causal relationships. Mechanistic interpretations should be regarded as hypothesis-generating and require validation through studies that integrate longitudinal epidemiological data with genetic and immune profiling.

## 9 Conclusion

Several immune-mediated diseases, including autoimmune thyroid disease, T1D, psoriasis, and IBD, have been found to frequently co-occur with MS. Separate studies reported increased occurrence of selected immune-mediated diseases following recorded MS diagnosis, while some previously diagnosed immune-mediated diseases were associated with subsequently diagnosed MS. However, the direction, magnitude, precision, and consistency of these findings varied across diseases, populations, study designs, and temporal frameworks. Evidence regarding associations with MS activity, disability, and progression remains limited and heterogeneous. These associations may indicate shared susceptibility, but could also result from surveillance bias, diagnostic delay, treatment exposure, residual confounding, or coincidental coexistence. Current evidence does not demonstrate that these conditions constitute an MS prodrome, share a unified immune architecture, or define biologically distinct MS subgroups. Future longitudinal studies should use standardized disease definitions, clearly documented diagnostic chronology, select appropriate comparator populations, incorporate treatment exposure effects, and adequately adjust for relevant confounders. In addition, mechanistic studies are required to clarify whether shared genetic or immunological factors contribute to the observed associations and to assess the clinical or prognostic significance of these associations.

## Data Availability

The original contributions presented in the study are included in the article/**Supplementary Material**. Further inquiries can be directed to the corresponding author.
