# Supplementary material for: Immune-mediated comorbidities in multiple sclerosis: a scoping review
Source: Front Immunol. 2026 Oct 28;17:1911169. doi: 10.3389/fimmu.2026.1911169 (PMC13751426; doi:10.3389/fimmu.2026.1911169)
Supplement: Supplementary file 1 [file Table1.docx]

**Supplementary Table S1. Specific search strategies used in PubMed, Elsevier Embase, and Scopus.**

**PubMed (Number of records: 436; Search date: Feb 28, 2026)**

("Multiple Sclerosis"[Mesh] OR "multiple sclerosis"[tiab] OR "disseminated sclerosis"[tiab])

AND

("Autoimmune Diseases"[Mesh] OR "autoimmune disease*"[tiab] OR "autoimmune disorder*"[tiab] OR "immune-mediated disease*"[tiab] OR "immune mediated disease*"[tiab] OR "immune-mediated disorder*"[tiab] OR "immune mediated disorder*"[tiab] OR "Inflammatory Bowel Diseases"[Mesh] OR "inflammatory bowel disease*"[tiab] OR IBD[tiab] OR "Crohn Disease"[Mesh] OR "Crohn disease"[tiab] OR "Crohn's disease"[tiab] OR "Colitis, Ulcerative"[Mesh] OR "ulcerative colitis"[tiab] OR "Psoriasis"[Mesh] OR psoria*[tiab] OR "Arthritis, Rheumatoid"[Mesh] OR "rheumatoid arthritis"[tiab] OR "Lupus Erythematosus, Systemic"[Mesh] OR "systemic lupus erythematosus"[tiab] OR SLE[tiab] OR "Diabetes Mellitus, Type 1"[Mesh] OR "type 1 diabetes"[tiab] OR "type I diabetes"[tiab] OR T1D[tiab] OR "Thyroid Diseases"[Mesh] OR "Thyroiditis, Autoimmune"[Mesh] OR "thyroid disease*"[tiab] OR "autoimmune thyroid*"[tiab] OR thyroiditis[tiab] OR Hashimoto*[tiab] OR "Graves disease"[tiab] OR "Asthma"[Mesh] OR asthma*[tiab] OR "Dermatitis, Atopic"[Mesh] OR "atopic dermatitis"[tiab] OR eczema[tiab] OR atop*[tiab] OR allerg*[tiab] OR "Vitiligo"[Mesh] OR vitiligo[tiab] OR "Celiac Disease"[Mesh] OR "celiac disease"[tiab] OR "coeliac disease"[tiab])

AND

("Comorbidity"[Mesh] OR "Prevalence"[Mesh] OR "Incidence"[Mesh] OR "Risk Factors"[Mesh] OR "Disease Progression"[Mesh] OR "Disability Evaluation"[Mesh] OR "Magnetic Resonance Imaging"[Mesh] OR comorbid*[tiab] OR prevalence[tiab] OR incidence[tiab] OR epidemiolog*[tiab] OR risk[tiab] OR association*[tiab] OR coexist*[tiab] OR co-occur*[tiab] OR "disease activity"[tiab] OR "disease course"[tiab] OR disability[tiab] OR progression[tiab] OR relapse*[tiab] OR MRI[tiab] OR "magnetic resonance imaging"[tiab] OR "brain volume*"[tiab])

AND

("2001/01/01"[dp] : "2026/02/28"[dp])

AND

English[Language]

**Elsevier Embase (Number of records: 612; Search date: Feb 28, 2026)**

('multiple sclerosis'/exp OR 'multiple sclerosis':ti,ab,kw OR 'disseminated sclerosis':ti,ab,kw)

AND

('autoimmune disease'/exp OR 'autoimmune disease*':ti,ab,kw OR 'autoimmune disorder*':ti,ab,kw OR 'immune-mediated disease*':ti,ab,kw OR 'immune mediated disease*':ti,ab,kw OR 'immune-mediated disorder*':ti,ab,kw OR 'immune mediated disorder*':ti,ab,kw OR 'inflammatory bowel disease'/exp OR 'inflammatory bowel disease*':ti,ab,kw OR ibd:ti,ab,kw OR 'crohn disease'/exp OR 'crohn disease':ti,ab,kw OR 'crohn''s disease':ti,ab,kw OR 'ulcerative colitis'/exp OR 'ulcerative colitis':ti,ab,kw OR 'psoriasis'/exp OR psoria*:ti,ab,kw OR 'rheumatoid arthritis'/exp OR 'rheumatoid arthritis':ti,ab,kw OR 'systemic lupus erythematosus'/exp OR 'systemic lupus erythematosus':ti,ab,kw OR sle:ti,ab,kw OR 'type 1 diabetes mellitus'/exp OR 'type 1 diabetes':ti,ab,kw OR 'type i diabetes':ti,ab,kw OR t1d:ti,ab,kw OR 'thyroid disease'/exp OR 'autoimmune thyroiditis'/exp OR 'thyroid disease*':ti,ab,kw OR 'autoimmune thyroid*':ti,ab,kw OR thyroiditis:ti,ab,kw OR hashimoto*:ti,ab,kw OR 'graves disease':ti,ab,kw OR 'asthma'/exp OR asthma*:ti,ab,kw OR 'atopic dermatitis'/exp OR 'atopic dermatitis':ti,ab,kw OR eczema:ti,ab,kw OR atop*:ti,ab,kw OR allerg*:ti,ab,kw OR 'vitiligo'/exp OR vitiligo:ti,ab,kw OR 'celiac disease'/exp OR 'celiac disease':ti,ab,kw OR 'coeliac disease':ti,ab,kw)

AND

('comorbidity'/exp OR 'prevalence'/exp OR 'incidence'/exp OR 'risk'/exp OR 'epidemiology'/exp OR 'disease course'/exp OR 'disease progression'/exp OR 'disability'/exp OR 'relapse'/exp OR comorbid*:ti,ab,kw OR prevalence:ti,ab,kw OR incidence:ti,ab,kw OR epidemiolog*:ti,ab,kw OR risk:ti,ab,kw OR association*:ti,ab,kw OR coexist*:ti,ab,kw OR 'co-occur*':ti,ab,kw OR 'disease activity':ti,ab,kw OR 'disease course':ti,ab,kw OR disability:ti,ab,kw OR progression:ti,ab,kw OR relapse*:ti,ab,kw OR mri:ti,ab,kw OR 'magnetic resonance imaging':ti,ab,kw OR 'brain volume*':ti,ab,kw)

AND

[2001-2026]/py

AND

english:la

**Scopus (Number of records: 520; Search date: Feb 28, 2026)**

TITLE-ABS-KEY("multiple sclerosis" OR "disseminated sclerosis")

AND

TITLE-ABS-KEY("autoimmune disease*" OR "autoimmune disorder*" OR "immune-mediated disease*" OR "immune mediated disease*" OR "immune-mediated disorder*" OR "immune mediated disorder*" OR "inflammatory bowel disease*" OR IBD OR "Crohn disease" OR "Crohn's disease" OR "ulcerative colitis" OR psoria* OR "rheumatoid arthritis" OR "systemic lupus erythematosus" OR SLE OR "type 1 diabetes" OR "type I diabetes" OR T1D OR "thyroid disease*" OR "autoimmune thyroid*" OR thyroiditis OR Hashimoto* OR "Graves disease" OR asthma* OR "atopic dermatitis" OR eczema OR atop* OR allerg* OR vitiligo OR "celiac disease" OR "coeliac disease")

AND

TITLE-ABS-KEY(comorbid* OR prevalence OR incidence OR epidemiolog* OR risk OR association* OR coexist* OR "co-occur*" OR "disease activity" OR "disease course" OR disability OR progression OR relapse* OR MRI OR "magnetic resonance imaging" OR "brain volume*")

AND

PUBYEAR > 2000

AND

PUBYEAR < 2027

AND

LANGUAGE(english)
